# Rhinovirus 3C protease suppresses apoptosis and triggers caspase-independent cell death

**DOI:** 10.1038/s41419-018-0306-6

**Published:** 2018-02-15

**Authors:** Mark Lötzerich, Pascal S. Roulin, Karin Boucke, Robert Witte, Oleg Georgiev, Urs F. Greber

**Affiliations:** 1 Department of Molecular Life Sciences, University of Zurich, Winterthurerstrasse 190, 8057 Zurich, Switzerland; 2 Present Address: Hussman Institute for Autism, 801 West Baltimore Street, Baltimore, MD 21201 USA

## Abstract

Apoptosis and programmed necrosis (necroptosis) determine cell fate, and antagonize infection. Execution of these complementary death pathways involves the formation of receptor-interacting protein kinase 1 (RIPK1) containing complexes. RIPK1 binds to adaptor proteins, such as TRIF (Toll-IL-1 receptor-domain-containing-adaptor-inducing interferon-beta factor), FADD (Fas-associated-protein with death domain), NEMO (NF-κB regulatory subunit IKKγ), SQSTM1 (sequestosome 1/p62), or RIPK3 (receptor-interacting protein kinase 3), which are involved in RNA sensing, NF-κB signaling, autophagosome formation, apoptosis, and necroptosis. We report that a range of rhinoviruses impair apoptosis and necroptosis in epithelial cells late in infection. Unlike the double-strand (ds) RNA mimetic poly I:C (polyinosinic:polycytidylic acid), the exposure of dsRNA to toll-like receptor 3 (TLR3) in rhinovirus-infected cells did not lead to apoptosis execution. Accordingly, necroptosis and the production of ROS (reactive oxygen species) were not observed late in infection, when RIPK3 was absent. Instead, a virus-induced alternative necrotic cell death pathway proceeded, which led to membrane rupture, indicated by propidium iodide staining. The impairment of dsRNA-induced apoptosis late in infection was controlled by the viral 3C-protease (3Cpro), which disrupted RIPK1-TRIF/FADD /SQSTM1 immune-complexes. 3Cpro and 3C precursors were found to coimmuno-precipitate with RIPK1, cleaving the RIPK1 death-domain, and generating N-terminal RIPK1 fragments. The depletion of RIPK1 or chemical inhibition of its kinase at the N-terminus did not interfere with virus progeny formation or cell fate. The data show that rhinoviruses suppress apoptosis and necroptosis, and release progeny by an alternative cell death pathway, which is controlled by viral proteases modifying innate immune complexes.

## Introduction

Apoptosis and necroptosis control the fate of selected cells during development of multicellular organisms. They are distinct hallmarks of host defense against pathogens, and tune the immunological tolerogenic or immunogenic responses^1–4^. Cells dying by apoptosis condense chromatin and disperse into membrane-wrapped fragments, whereas necrotic cells release their contents and elicit innate immune responses from immune and non-immune cells. Apoptosis requires proteolysis by caspases, and phenotypically involves blebbing of the plasma membrane, and nuclear DNA fragmentation without cell lysis^5,6^. Necrosis does not require caspases, and leads to cell swelling, membrane rupture, and leakage of cytoplasm^1^. Programmed necrosis is known as necroptosis, and has important roles in development. Apoptosis and necroptosis can be triggered by activation of Toll-like receptors (TLR), or virus infection^4,7^.

RNA viruses can set off cell death through DNA damage or production of double-strand RNA (dsRNA), activation of TLR3, retinoic acid inducible gene I (RIG-I)-like receptors (RLR), protein kinase R (PKR), or indirectly through extrinsic pathways, such as tumor necrosis factor receptor (TNFR) signaling. They antagonize cell death pathways by dedicated proteins, and thereby tune the production and release of virions from infected cells^8–10^. Picornaviruses, such as poliovirus (PV), coxsackievirus (CV) or encephalomyocarditis virus (EMCV) are thought to induce apoptosis but also to inhibit apotosis execution^8,11–17^. In addition, picornavirus infection may interfere with innate immunity related IFN-signaling^17–20^.

Mechanisms of cell death of rhinovirus (RV)-infected cells are unknown. Human RVs (HRVs) belong to the Enterovirus genus of the *Picornaviridae*. They are the causative agents of the common cold, triggering mild symptoms in many individuals. In individuals with asthma, chronic obstructive pulmonary disease or cystic fibrosis HRV infections have severe and often life-threatening complications^21^. This is associated with altered integrity of respiratory epithelia, and innate and adaptive immune responses^22^. HRV trigger innate immunity reactions upon replication on cytoplasmic tubulo-vesicular membranes of epithelial cells in the upper respiratory tract, due to danger signals, such as viral dsRNA intermediates^23–25^. Danger signals from enteroviruses are decoded by TLR3 and the RNA helicase MDA5 (melanoma differentiation-associated gene 5), which trigger an innate anti-viral response^26–28^. Such response can lead to apoptosis and eliminate infected cells without largely affecting integrity of upper respiratory tracts^16,22^. At exacerbated conditions, lower respiratory tract infections are more destructive due to induction of unknown immune-stimulatory cell death pathways^21^.

Enteroviruses target TLR3, MDA5 and the transducers TRIF (Toll-IL-1 receptor-domain-containing-adaptor-inducing interferon-beta factor) and MAVS (mitochondrial antiviral signaling protein) by their proteases 2A and 3C, or indirectly by caspase activation, and attenuate pro-inflammatory cytokine and type I interferon production^2,18,29,30^. TLR3-signaling is not only associated to proinflammatory cytokine response but also to apoptotic- and necroptotic cell death. In epithelial cells viral dsRNA signaling involving TLR3 induces caspase-8-mediated apoptosis that depends on RIPK1 and TRIF. Receptor-interacting serine/threonine-protein kinase 1 (RIPK1) is highly conserved in vertebrates and essential for organismic homeostasis^31–33^. It forms signaling complexes controlling execution of apoptosis or necroptosis^2,4,7,34–36^. Its N-terminal kinase domain is important for necroptotic cell death. The intermediate domain recruits adaptor proteins including p62/SQSTM1, and NEMO (NF-kappa B essential modulator), for regulation of cell death, autophagy, and inflammation^37^. The RIP-homotypic interaction motif (RHIM) binds to the TLR3/TLR4 adaptor TRIF and RIPK3. The C-terminal death domain (DD) enables interactions with death receptors TNFR1, Fas, TRAIL-R1/R2 and adaptors, such as FADD (Fas-associated protein with DD) or TRADD (TNF-receptor-associated death domain). The absence of caspase activity redirects extrinsic death pathways from apoptosis towards necroptosis^7,33,38^. Here we investigated how rhinoviruses target RIPK1 to toggle-switch between apoptosis and necroptosis, and control cell death pathways.

## Results

### HRV infection induces necrosis rather than apoptosis in primary cells and cell cultures

**Fig. 1:**
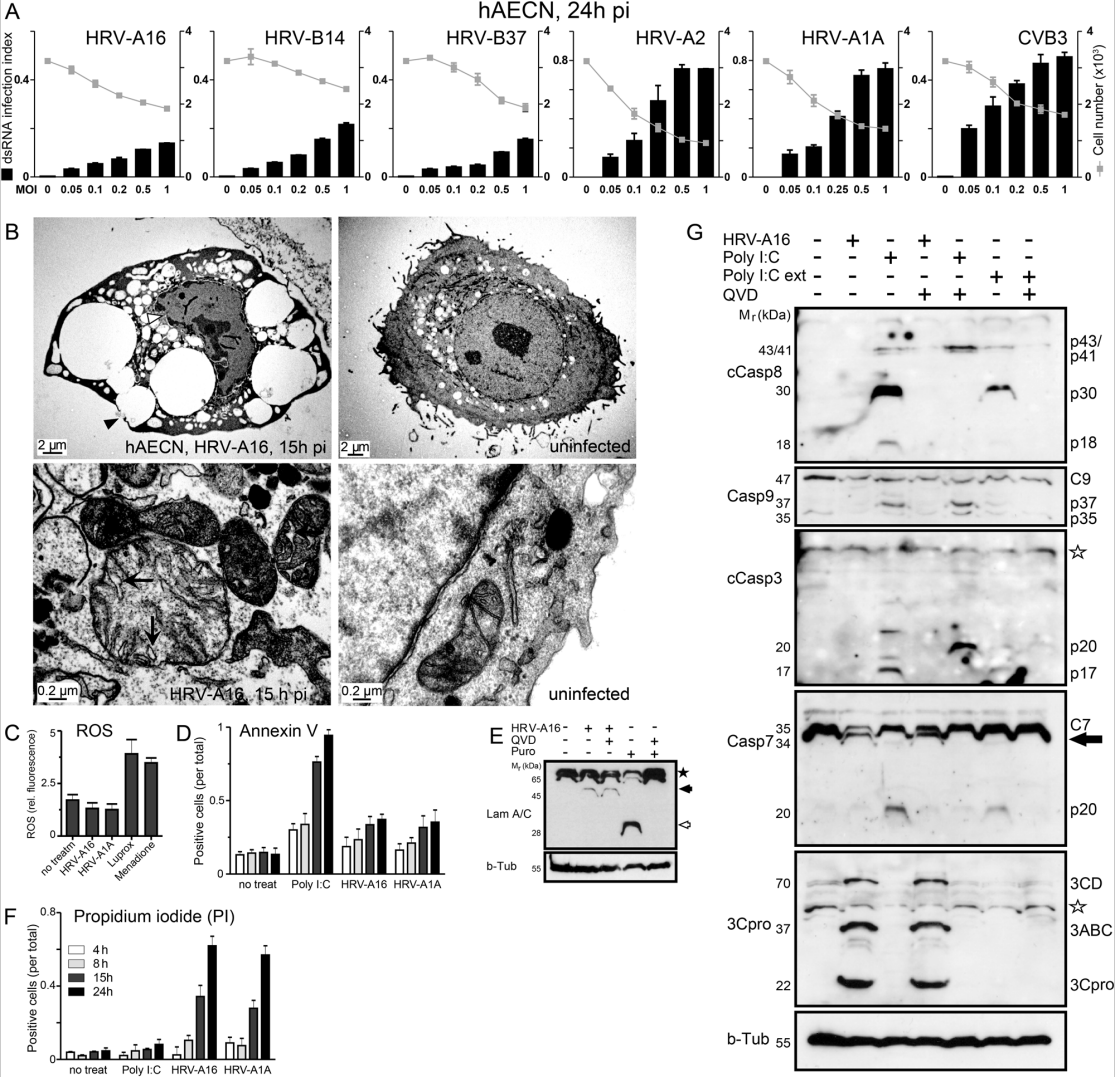
Necrotic cell death rather than apoptosis in HRV-A16-infected primary human nasal epithelial cells. **a** Dose-dependent infection of hAECN with various A- and B-type rhinoviruses (HRV-A16, HRV-B14, HRV-B37, HRV-A2, HRV-A1A), and CVB3 and correlation with cell number. Values are means ± SD, *n* = 3. **b** Electron micrographs of hAECN infected with HRV-A16 (MOI 1) or treated with poly I:C or puromycin for 15 h, or uninfected cells. Proximity of vesicular structures with the cell surface indicated by black arrowheads, and swollen mitochondrial cristae in infected cells highlighted by arrows. **c** ROS production of HRV-A16 or HRV-A1A (MOI 1) infected cells 24 h pi, compared to luprox- (100 μm, 1 h) or menadione (100 μm, 1 h)-treated cells. **d** FACS analysis of annexin V stainings of hAECN infected with HRV-A16 or HRV-A1A (MOI 1) for 4, 8, 15 or 24 h and comparison with poly I:C-treated cells. Values are means from approximately 10,000 cells analyzed ± SD, *n* = 3. **e** Western blots against lamin A/C and beta-tubulin from lysates of hAECN cells infected with HRV-A16 (MOI 1, 15 h) or treated with puromycin (5 µg/ml, 15 h) with or without pan-caspase inhibitor QVD (5 µm). *M*_r_ denotes relative molecular weight in kDa. **f** FACS analysis propidium iodide (PI) stainings of hAECN infected with HRV-A16 or HRV-A1A (MOI 1) for 4, 8, 15 or 24 h and comparison with poly I:C-treated cells. Values are means from approximately 10,000 cells analyzed ± SD, *n* = 3. **g** Western blot analysis of caspase 3, 7, 8, 9 activations in hAECN after infection with HRV-A16 (MOI 1, 15 h), poly I:C transfection (5 μg/ml) or external poly I:C addition (ext, 50 μg/ml). Arrow highlights virus-induced caspase-7 processing product; undefined antibody background is indicated by a star. Infection is indicated by blotting against 3Cpro using beta-tubulin as a loading control.

**Fig. 2:**
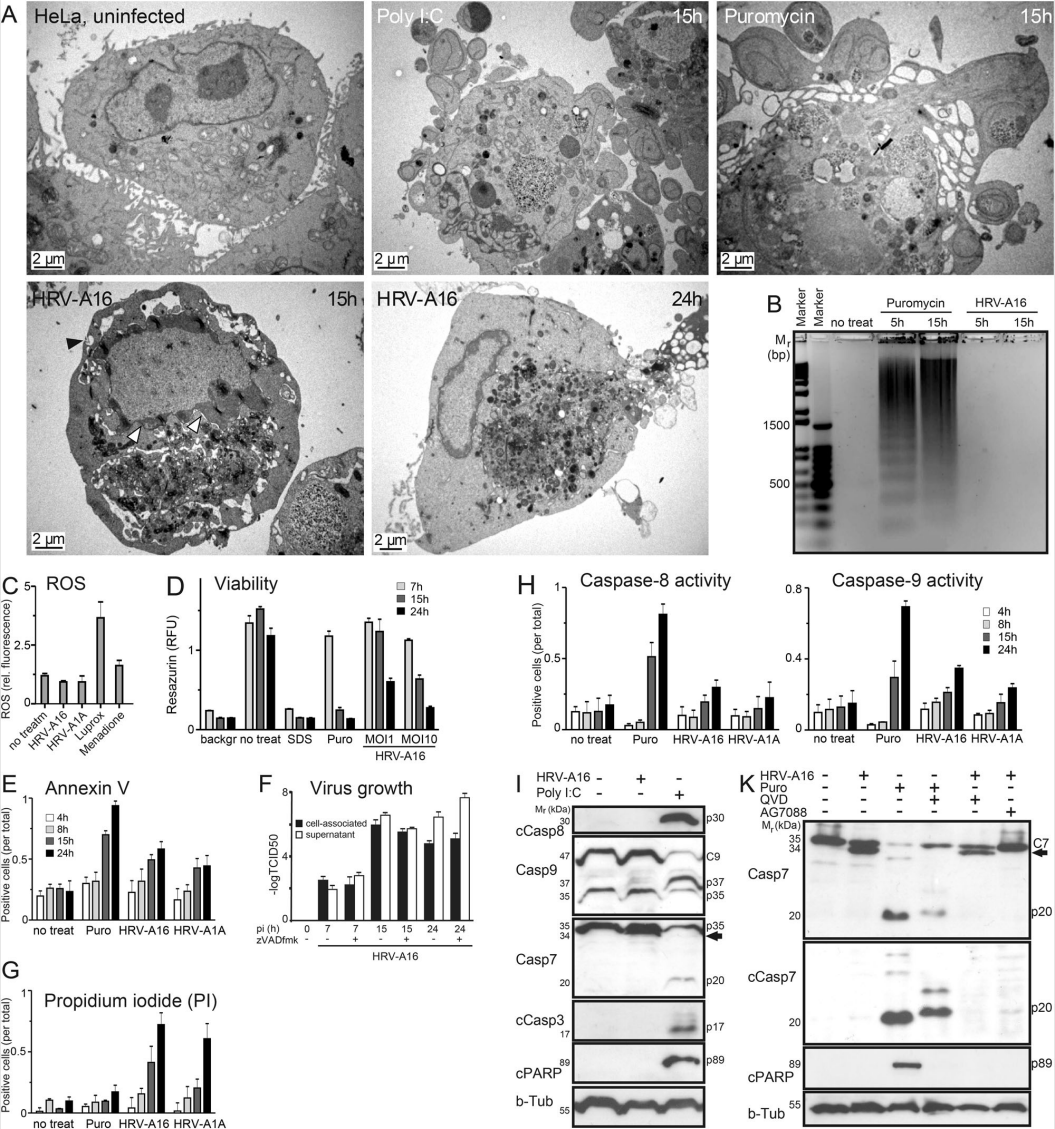
Rhinovirus induces cell death without apoptotic signaling. **a** Electron micrographs of HeLa cells infected with HRV-A16 (MOI 1) or treated with poly I:C or puromycin for 15 h, or uninfected cells. Proximity of vesicular structures with the cell surface indicated by black arrowheads, and swollen mitochondrial cristae in infected cells highlighted by arrows. **b** Genomic DNA-laddering assay of HeLa cells infected with HRV-A16 in comparison with puromycin-treated cells. *M*_r_ denotes relative DNA length in base pairs (bp). **c** ROS production of HRV-A16- or HRV-A1A (MOI 1)-infected cells 24 h pi, compared to luprox- (100 μm, 1 h) or menadione (100 μm, 1 h)-treated HeLa cells. **d** Resazurin viability assay-measurement of metabolic activity on HeLa Ohio cells after treatment with SDS, puromycin or infection with HRV-A16 (MOI 1, 10) after different time points (7, 15, 24 h). Values are means ± SD, *n* = 6. **e** FACS analysis of extracellular annexin V of HeLa cells infected with HRV-A16 or HRV-A1A (MOI 1), and comparison with puromycin-treated cells. Values are means ± SD, *n* = 3. **f** HRV progeny formation is not influenced by pan-caspase inhibitors. TCID50 values of cell-associated and supernatant HRV-A16 (MOI 1) of an infection kinetics from HeLa cells infected with or without pan-caspase inhibitor zVADfmk (20 μm). Values are means ± SD, *n* = 3. **g** FACS analysis of propidium iodide (PI) stainings of HeLa cells infected with HRV-A16 or HRV-A1A (MOI 1), and comparison with puromycin-treated cells. Values are means ± SD, *n* = 3. **h** FLICA caspase-8/9 activity assays in HeLa cells infected with HRV-A16 (MOI 1) or HRV-A1A (MOI 1), and comparison with puromycin-treated cells. Values are means ± SD, *n* = 3. **i** Western blot analysis of caspase activation and PARP cleavage in HeLa cells after infection with HRV-A16 (MOI 1, 15 h) or poly I:C treatment. Small “c” denotes cleaved protein. **k** Western blots against caspase-7 (Casp7), cleaved caspase-7 (cCasp7), cleaved PARP (c-PARP), and beta-tubulin (b-Tub) from HeLa cells infected with HRV-A16 (MOI 1, 15 h), infected cells treated with pan-caspase inhibitor QVD (5 μm, 15 h), or AG7088 (rupintrivir, 20 nm, 15 h) and comparison with samples from puromycin- and QVD-treated cells. Arrow highlights the virus-induced caspase-7 processing product.

Human primary airway epithelial cells of nasal origin (hAECN), or HeLa cells, were infected with a panel of human rhinoviruses, HRV-A1A, A2, A16, B14, B37, and coxsackie virus B3 (CVB3) (Figs. 1a, 2). At an MOI of 1 all of these infections gave rise to virus-specific double strand (ds) RNA as early as 5–7 h post infection (pi), indicative of initial stages of viral replication^23,24,39^. HRV-A16 infection phenotypes were then analyzed by electron microscopy, and indicated necrotic features at 15 h pi, including rounding up, vacuolated cytoplasm, a convoluted nuclear envelope (white arrow heads), apparent plasma membrane bursts (black arrow heads), and alterations of the mitochondrial architecture, including swelling of the cristae without apparent membrane bursting (arrows, Figs. 1b, 2a). These phenotypes were distinct from apoptotic features induced by puromycin or the synthetic dsRNA mimetic poly I:C (poly-ribo-inosinic: poly-ribo-cytidylic acid) that induced the formation of apoptotic bodies (Fig. 2a). The results with puromycin and poly I:C were in agreement with previous reports^40,41^.

The absence of apoptotic features was reinforced by the observation that HRV-A16-infected HeLa cells showed no signs of inter-nucleosomal cleavage of DNA by nucleases into multiples of 180 bp fragments, unlike puromycin-treated cells (Fig. 2b). Infected cells showed no signs of unspecific DNA processing, indicative of necrotic cell death nor reactive oxygen species (ROS), and no reduction in metabolic activity, except very late in infection (Figs. 1c, d; 2a−d). Poly I:C and puromycin treatment of hAECN and HeLa cells generated a strong annexin V signal indicating apoptosis. In contrast, infected cells became only slightly annexin V-positive late in infection (Figs. 1d, 2e). Poly I:C or puromycin- induced apoptosis was inhibited by pan-caspase inhibitors QVD or zVADfmk but not by the necroptosis inhibitor necrostatin 1 (NEC-1) or 3Cpro inhibitor AG7088 (Supplementary Figure S1A−B). Puromycin treatment of hAECN cells induced another hallmark of apoptosis, the cleavage of lamin A/C, which could be inhibited by addition of the pan-caspase inhibitor Q-VD-OPH (QVD, Fig. 1e). HRV-A16 infection led to distinct lamin A/C cleavage at late time points, independent of QVD, suggesting caspase-independent cleavage (Fig. 1e). Notably, the inhibition of apoptosis by the pan-apoptosis inhibitor zVADfmk did not affect the growth of HRV-A16 (Fig. 2f).

Significantly, the HRV-A1A or A16-infected hAECN or HeLa cells became strongly positive for propidium iodide (PI), unlike poly I:C-treated cells, indicating the loss of plasma membrane integrity at 15 h pi (Figs. 1f, 2g). Puromycin- or poly I:C-induced cell leakage was enhanced by QVD and z-VADfmk, and not affected by NEC-1 or AG7088 (Rupintrivir)^42^, validating the specificity of the assay systems (Supplementary Figure S1C−D). Enzymatic FLICA caspase-8/9 assays showed low levels of activation of initiator caspases at 15 or 24 h pi, but robust levels in puromycin-treated cells (Fig. 2h, and Supplementary Figure S2). This was confirmed by western blotting for cleaved caspase-8 (Supplementary Figure S1E). The treatment of hAECN or HeLa cells with poly I:C generated a procaspase-8 cleavage product p30 (Figs. 1g,  2i), which had been described to conduct an alternative pathway of apoptosis^43^. It also stimulated intrinsic apoptosis, as indicated by the p37 processing product of procaspase-9. In contrast, no active p30 and very little p37 fragments were detectable in HRV-A16-infected cells (Figs. 1g, 2i). The caspase-8/9 activations were validated by enzymatic activity assays using caspase-8/9 inhibitors z-IETDfmk and z-LEHDfmk (Supplementary Fig. S2A−B). In accordance, we found no activation of caspase-3/7 or cleavage of PARP in HRV-A16-infected cells, whereas puromycin or poly I:C treatment led to activation of the effector caspases-3/7 (p17 and p20 fragments), and cleavage of the caspase substrate Poly-ADP-ribose polymerase (PARP) (see 89 kDa fragment in Figs. 1g, 2i,k). Interestingly, caspase-7 was cleaved to a p34 fragment in the infected cells, insensitive to QVD, unlike in puromycin-treated cells (Figs. 1g, 2k). The appearance of p34 was, however, inhibited by AG7088, suggesting that caspase-7 is a substrate for proteases of infected cells (Fig. 2k).

Taken together, the data suggest that although dsRNA is abundantly produced during HRV-A16 infection, and although apoptosis is induced in HeLa or hAECN cells by synthetic dsRNA, no signs of apoptosis execution were evident in the infected cells. Yet, late in infection (24 h pi), the cells displayed necrotic rather than apoptotic features. Virus growth and release were insensitive to pan-caspase inhibitors.

### Rhinovirus infection suppresses the pro-apoptotic TLR3 pathway

**Fig. 3:**
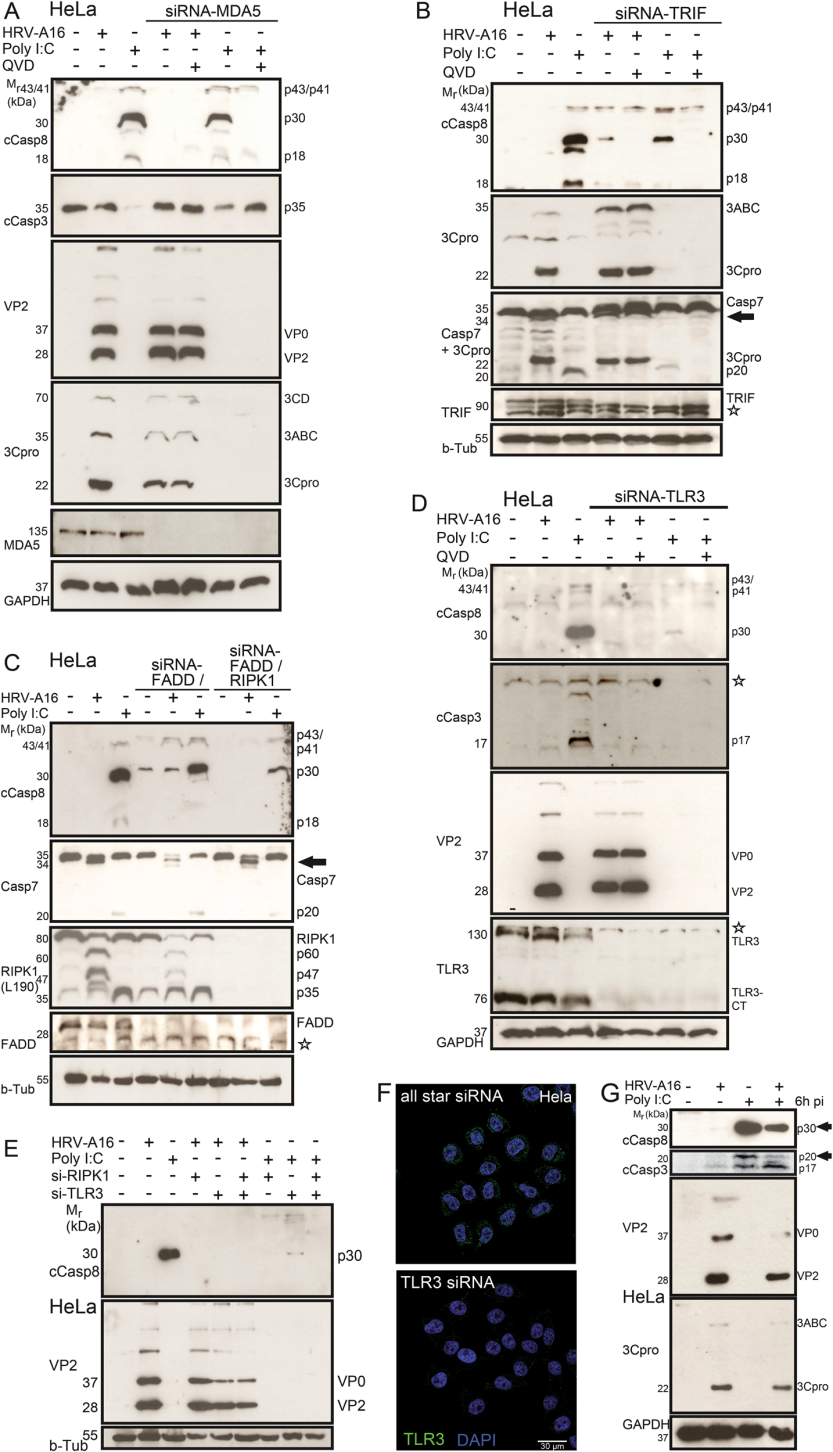
TLR3 is required for poly I:C-induced caspase-8 activation, which can be suppressed by HRV-A16 infection, while MDA5, TRIF, and FADD are dispensable for caspase-8 activation. **a** Western blots against cleaved caspase-8 (cCasp8), cleaved caspase-3 (cCasp3), VP2, 3Cpro, MDA5, and GAPDH from lysates of HeLa cells treated with siRNA against MDA5 and infected with HRV-A16 (MOI 1, 15 h), transfected with poly I:C (5 μg/ml) or untreated samples, and comparison of protein expression after siRNA-MDA5 treatment ± pan-caspase inhibitor QVD. **b** Western blots against cleaved caspase-8 (cCasp8), 3Cpro, caspase-7 (Casp7 + 3Cpro serial antibody incubation), TRIF, and beta-tubulin from lysates of HeLa cells infected with HRV-A16 (MOI 1, 15 h), transfected with poly I:C (5  μg/ml) or untreated samples and comparison of protein expression after siRNA-TRIF treatment ± pan-caspase inhibitor QVD; black arrow highlights virus-induced caspase-7 processing product; undefined antibody background is indicated by a star. **c** Western blots against cleaved caspase-8 (cCasp8), caspase-7 (Casp7), RIPK1 (aa190), FADD, and beta-tubulin from lysates of HeLa cells infected with HRV-A16 (MOI 1, 15 h), transfected with poly I:C (5 μg/ml) or untreated samples, and comparison of protein expression after siRNA-FADD and siRNA-FADD/RIPK1 treatment. Black arrow highlights virus-induced caspase-7 processing product; undefined antibody background is indicated by a star. **d** Western blots against cleaved caspase-8 (cCasp8), cleaved caspase-3 (cCasp3), VP2, TLR3, and GAPDH from lysates of HeLa cells infected with HRV-A16 (MOI 1, 15 h), transfected with poly I:C (5 µg/ml) or untreated, and comparison of protein expression after siRNA-TLR3 treatment with or without QVD (5 µm). Stars indicate unspecific background staining. TLR3-CT denotes C-terminal cleaved form of TLR3. **e** Western blots of cleaved caspase-8 (cCasp8), VP2, and beta-tubulin from lysates of HeLa cells infected with HRV-A16 (MOI 1, 15 h), transfected with poly I:C (5 μg/ml) or untreated samples, and comparison of protein expression after siRNA-RIPK1, siRNA-TLR3 or siRNA-RIPK1/TLR3 treatment. **f** Knock-down of TLR3 by RNA interference in HeLa cells, analyzed by single section confocal fluorescence microscopy. Control transfections with all star siRNA are shown on the left. TLR3 immune-staining in green, nuclei (DAPI) in blue. Scale bar 30 µm. **g** HRV-A16 (MOI 1, 15 h)-mediated suppression of caspase-8 activation indicated by western blots against cleaved caspase-8 (cCasp8, arrow), cleaved caspase-3 (cCasp3, p17/p20, arrow), VP2, 3C, and GAPDH from lysates of HeLa cells after late addition of poly I:C (5 µg/ml, 6 h).

**Fig. 4:**
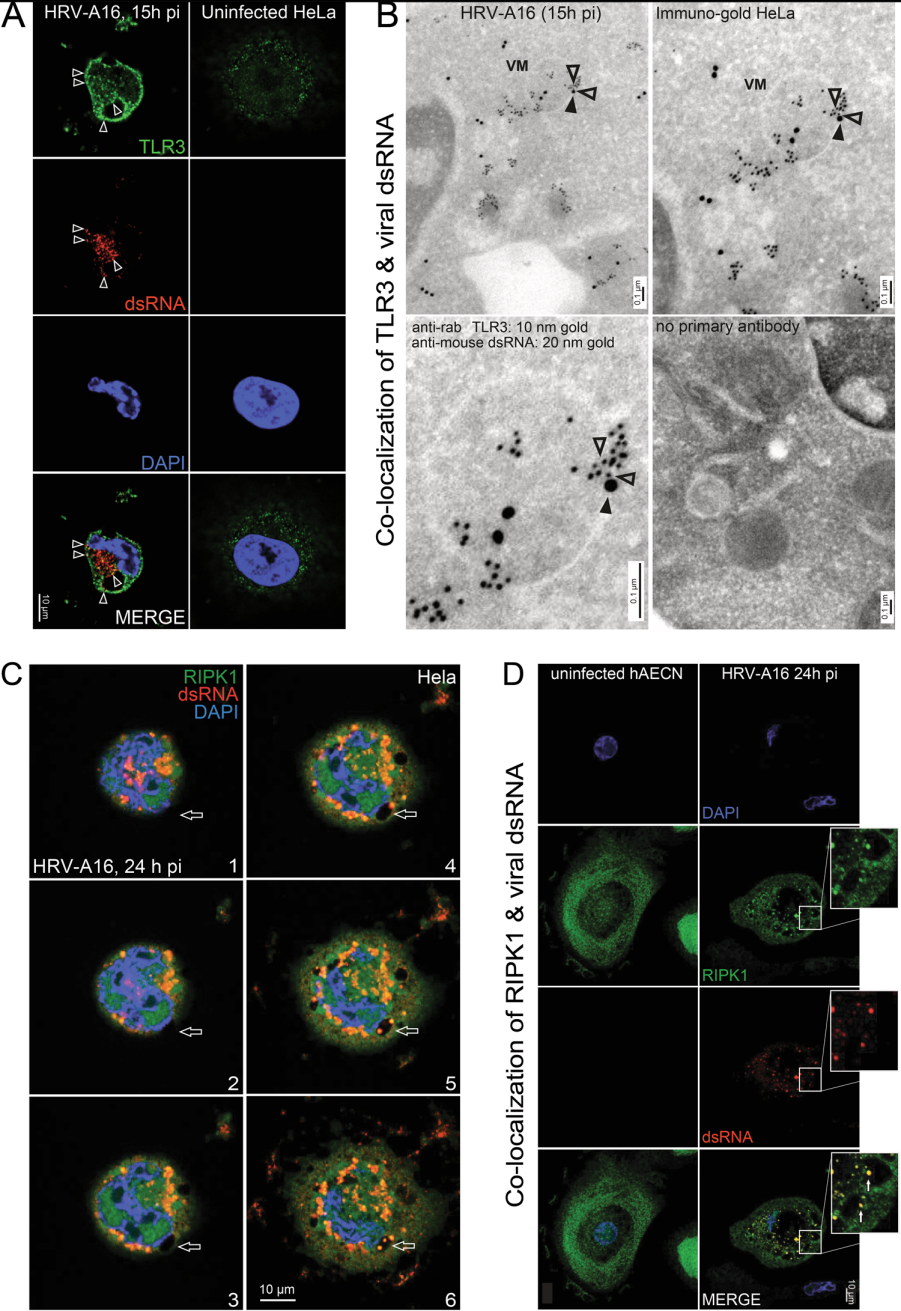
Colocalization of viral dsRNA with TLR3 and RIPK1. **a** Single section confocal microscopy images of uninfected HRV-A16 (MOI 1, 15 h)-infected HeLa cells stained for TLR3 (green), dsRNA (red), and nuclei (blue). White arrowheads point to structures positive for TLR3 and dsRNA. Scale bar 10 µm. **b** Tokuyasu cryo-immuno-sections of HeLa cells infected with HRV-A16 (MOI 1, 15 h); TLR3 was visualized with a rabbit TLR3 antibody and goat anti-rabbit IgG 10 nm immuno-gold-conjugated secondary antibody (open arrowheads). dsRNA was visualized with the Mab J2 and goat anti-mouse IgG 20 nm immuno-gold-conjugated secondary antibody (black arrowheads). Control section without immuno-gold-conjugated secondary antibody is shown in the lower right corner. VM denotes vesicular membrane. **c** Serial z-stacked confocal microscopy sections of HeLa cells infected with HRV-A16 (MOI 1, 15 h), stained for RIPK1 (green), dsRNA (red), and nuclei (DAPI, blue). Top section is 1, bottom section 6. Open arrows point to positions of vesicular structures double-positive for RIPK1 and dsRNA. Scale bar 10 µm. **d** Confocal microscopy images of hAECN infected with HRV-A16 (MOI 1, 24 h). Cells were stained for RIPK1 (green), dsRNA (red), and nuclei (blue). Single z-sections show colocalization of RIPK1 and dsRNA puncta (white arrows).

The dsRNA mimic poly I:C induces extrinsic and intrinsic apoptotic pathways through TLR3, MDA5, or PKR^44^. This was not affected by siRNA-mediated knockdown of MDA5 or FADD, unlike the knockdown of TLR3 and/or the TLR3 adaptor TRIF, which suppressed the activation of caspase-8, caspase-3, and caspase-7 (Fig. 3a−f). RIPK1 controls death signaling, and associates with TLR3, MDA5-MAVS, and PKR complexes to transmit dsRNA PAMP (pathogen-associated molecular pattern) signals reviewed in ref. ^45^. Knockdown of RIPK1 effectively suppressed activation of caspase-8 in poly I:C-treated cells (Fig. 3c, e). In contrast, HRV-A16-infected cells positive for dsRNA did not execute apoptosis. Fluorescence microscopy and immuno-gold cryo-sections revealed that a fraction of TLR3 localized to rhinovirus-induced dsRNA at large vesicular structures (Fig. 4a, b). The signal was specific since no TLR3 signal was observed in cells treated with TLR3-siRNAs (Fig. 3f). The dsRNA-positive structures also contained RIPK1, as demonstrated by single section confocal fluorescence microscopy in hAECN and HeLa cells (Fig. 4c, d). However, TLR3 or RIPK1 knockdown had essentially no effect on virus protein production (Fig. 3d, e). These results suggest that although dsRNAs might be accessed by TLR3 in the infected cells, the execution of apoptosis is suppressed.

This conclusion was strongly corroborated by the findings that HRV-A16 infection counteracted the activation and execution of apoptosis upon poly I:C or puromycin treatment. This notion was further supported by western blotting for cleaved caspase-8 (Fig. 3g), and annexin V assays (Supplementary Figure S2B−C, and S3A−B). Remarkably, HRV-A16 not only suppressed apoptosis but enhanced viral necrosis, as indicated by propidium iodide exclusion assays (Supplementary Figure 3C−D). Importantly, the expression of 3Cpro suppressed annexin V signals induced by poly I:C, whereas catalytically inactive 3Cpro induced apoptosis, albeit to a lesser extent than poly I:C (Supplementary Figure S4). These results support an important role of 3Cpro in suppressing apoptosis. On the other hand, 3Cpro, and to a lesser extent inactive 3Cpro induced cell lysis, as indicated by PI assays, whereas poly I:C had no significant effects on PI signals. These results establish that catalytically active 3Cpro suppresses apoptosis and enhances cell lysis in absence of other viral factors.

### Caspase-independent RIPK1 cleavage by rhinovirus

**Fig. 5:**
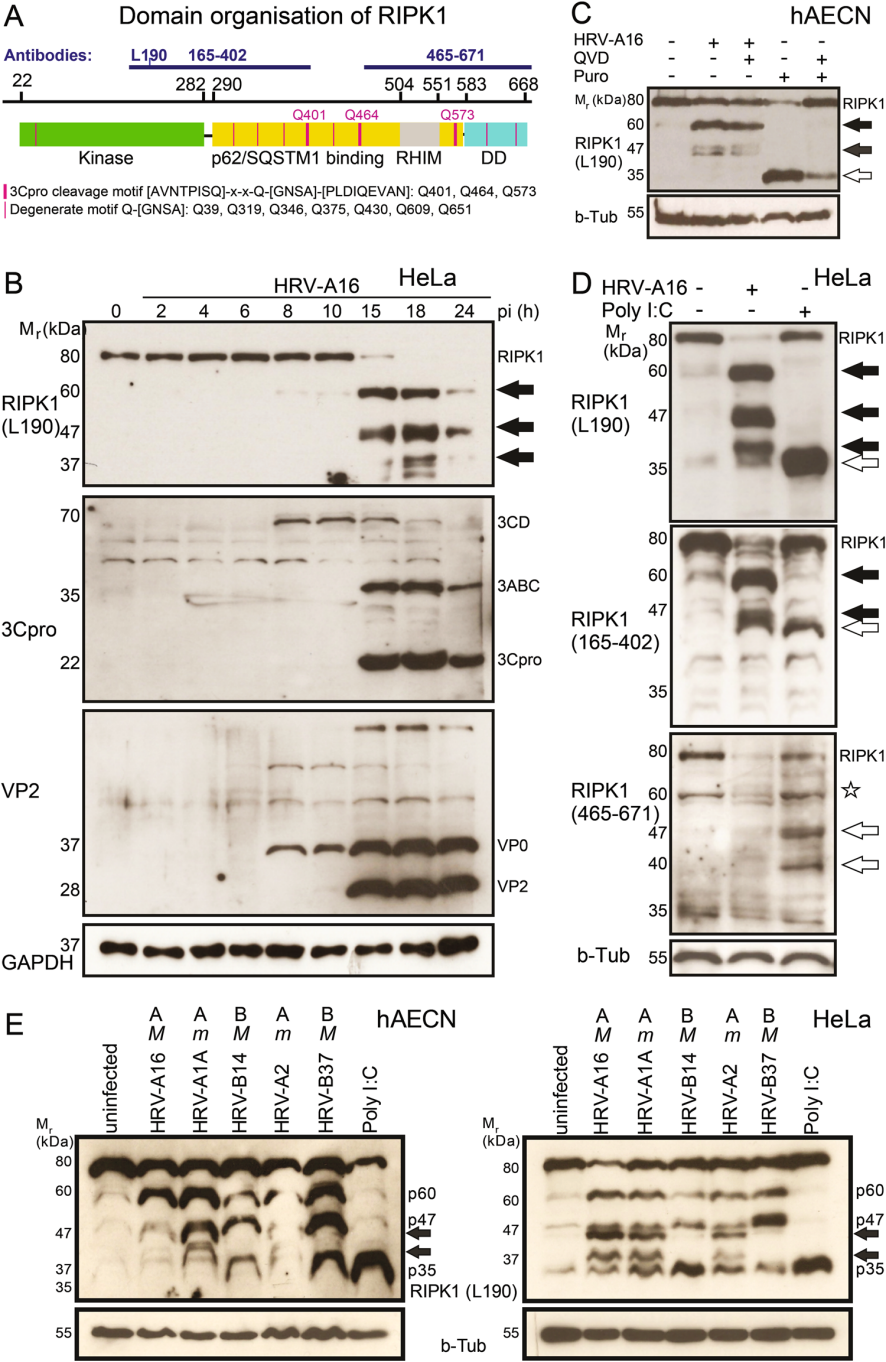
HRV-A16 induces caspase-independent cleavage of RIPK1. **a** Linear representation of human RIPK1 comprising amino acids 1–671. The kinase domain is depicted in green, the middle domain (yellow) comprising an RHIM domain (gray) is responsible for p62/SQSTM1 binding, and the C-terminal death domain (DD, turquoise) links RIPK1 to cell death pathways. The predicted 3C consensus sequences are marked by purple lines. Antibody binding domains are highlighted by blue lines and numbers. **b** Time-resolved analyses of HeLa cells infected with HRV-A16 (MOI 1) by western blots against N-terminal RIPK1 (aa190), VP2, 3C, and GAPDH. Virus-specific RIPK1 processing products are highlighted by black arrows, and viral precursor proteins 3CD, 3ABC, and VP0 are indicated on the right side. **c** Virus-specific RIPK1 processing in primary hAECN independent of caspase. Western blots are shown against RIPK1 (leucine 190) and beta-tubulin from lysates of hAECN cells infected with HRV-A16 (MOI 1, 15 h) or treated with puromycin (5 µg/ml, 15 h) with or without pan-caspase inhibitor QVD (5 µm). **d** Comparison of RIPK1 processing in infected and poly I:C-treated cells. Western blots against N-terminal RIPK1 (leucine 190), middle domain RIPK1 (amino acids 165–402), C-terminal RIPK1 (amino acids 465–671), and beta-tubulin from lysates of HeLa cells after HRV-A16 infection (MOI 1, 15 h) or poly I:C transfection (5 µg/ml, 15 h). N-terminal virus-induced products are highlighted by black arrows. Poly I:C-induced RIPK1 products are indicated by white arrows. Star represents an unidentified product also present in uninfected cells. **e** A range of rhinoviruses including A16, A1A, B14, A2, and B37 induces cleavage of RIPK1 in hAECN and HeLa cells. Western blots of lysates from infected (MOI 1, 15 h) or uninfected hAECN (left panel) or HeLa cells (right panel) probed with N-terminal anti-RIPK1 antibody (L190) and anti-beta tubulin (b-Tub) antibody. Capital “M” denotes major HRV types tropic for ICAM-1, small “m” denotes minor types tropic for LDLR and related proteins.

RIPK1 consists of 671 amino acids, with a central p62/SQSTM1 (sequestosome) binding domain, an RHIM-domain, and a C-terminal death-domain (DD) (Fig. 5a). Both RHIM and DD mediate RIPK1 binding to complexes composed of TRIF, RIPK3, FADD, TRADD, and caspase-8^46^. Inspection of the RIPK1 amino acid sequence showed that the middle p62/SQSTM1 binding domain contains three strictly defined HRV 3Cpro consensus motifs [AVNTPISQ]-x-x-Q-[GNSA]-[PLDIQEVAN] at Q401, 464, and 573^47,48^. The kinase, the middle and the C-terminal domains contain several degenerate Q-[GNSA] motifs.

Western blot analysis of HRV-A16-infected hAECN or HeLa cell lysates revealed efficient cleavage of the 80 kDa RIPK1 protein to 60 and 46/47 kDa proteins, both of which reacted with an antibody recognizing the N-terminal domain near leucine 190 (Fig. 5b, c). The 60 and 46/47 kDa proteins were prominent at 15 or 18 h pi when progeny was produced, as indicated by the presence of 3Cpro, VP2 and also virus titer (Fig. 5b, and data not shown). Additional N-terminal products were observed, including 36/37 kDa fragments, most likely processed at degenerate 3C consensus sites (Fig. 5b, c). In support of this notion, the 36/37 kDa fragments were not detected by an antibody recognizing epitopes between amino acids 165 and 402, although the antibody detected the 60 and the 46/47 kDa fragments (Fig. 5d). Neither the 60, the 46/47 nor the 36/37 kDa bands were recognized by an antibody against the C-terminal amino acids 465–671 comprising the RHIM and the DD domains (Fig. 5d). These results show that HRV-A16 infection generates N-terminal RIPK1 cleavage fragments comprising the kinase domain and variable amounts of the p62/SQSTM1 binding domain, but lacking the RHIM and DD domains. This virus-specific RIPK1 cleavage pattern was observed not only in HRV-A16- infected cells, but also in HRV-A1A, B14, A2, B37 but not poly I:C-treated hAECN or HeLa cells (Fig. 5e). Poly I:C treatment led to N-terminal proteolytic cleavage products migrating at 35 kDa, which were not recognized by the middle domain or the C-terminal RIPK1 antibodies (Fig. 5d). Poly I:C treatment further generated several C-terminal RIPK1 fragments of 45 and 40 kDa, which were not found in HRV-A16-infected cells.

### Rhinovirus 3Cpro cleaves RIPK1

**Fig. 6:**
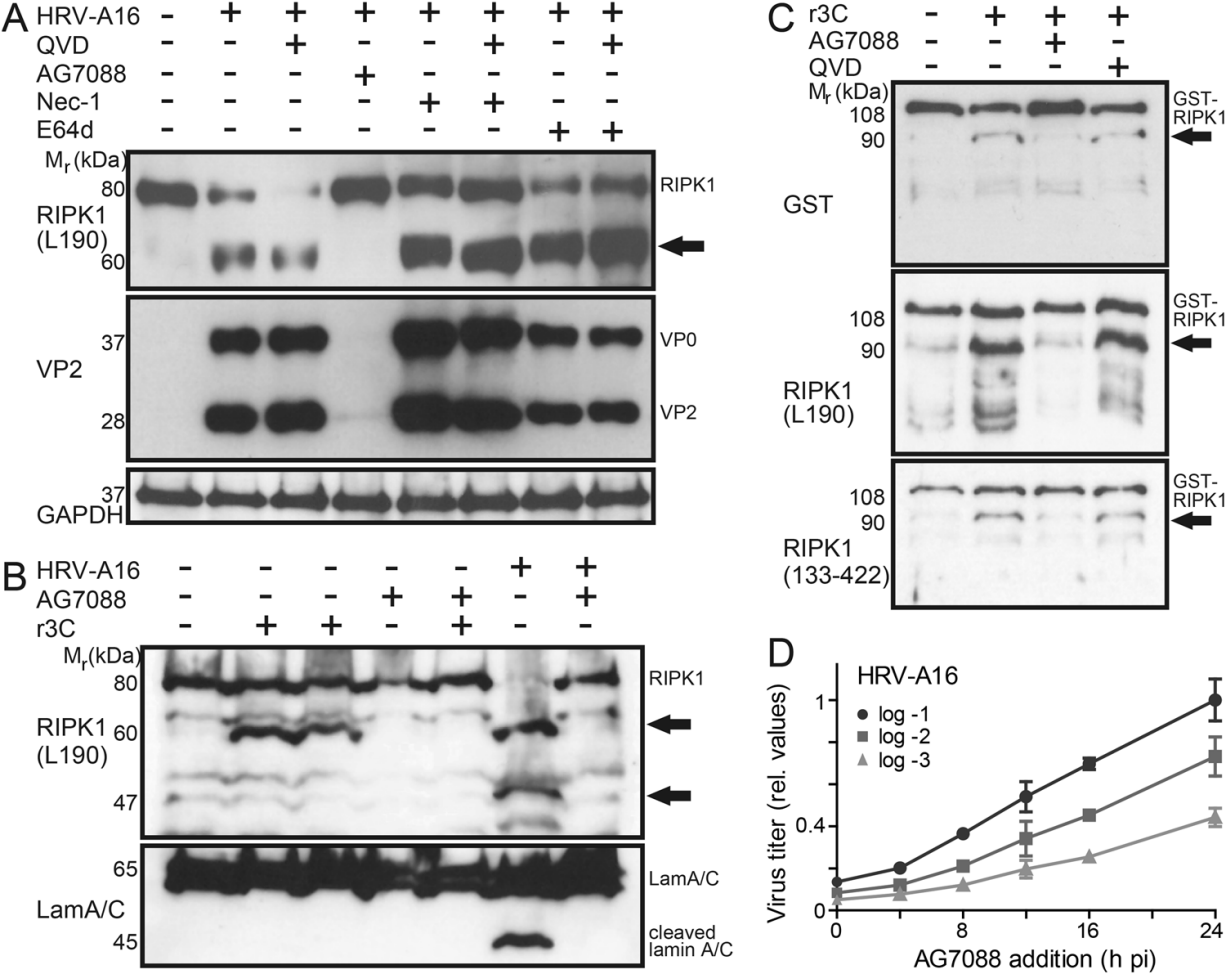
3Cpro generates a 60 kDa N-terminal RIPK1 fragment, and supports HRV-A16 titer production late in infection. **a** Western blots against RIPK1 (leucine 190), VP2, and GAPDH from lysates of HeLa cells infected with HRV-A16 (MOI 1, 15 h) and treated with pan-caspase inhibitor QVD (5 µm, 15 h), calpain/cathepsin inhibitor E64d (10 µm, 15 h), necrostatin-1 (Nec1, 1 µm, 15 h), AG7088 (rupintrivir, 20 nm, 15 h) or drug-combinations. The 60 kDa RIPK1 fragment is highlighted by black arrow. **b** In vitro cleavage of RIPK1 by 3Cpro. HeLa cell extracts were incubated with recombinant 3C protease (r3C) with or without the 3C inhibitor AG7088 (20 nm), and analyzed by western blotting against RIPK1 (leucine 190) and lamin A/C. Reference samples included lysates from HeLa infected with HRV-A16 (MOI 1, 15 h). RIPK1 cleavage is highlighted by black arrows. **c** GST-RIPK1 cleavage in vitro. Recombinant GST-RIPK1 was incubated with r3C in presence or absence of AG7088 (20 nm) or QVD (5 µm), and reaction products analyzed by western blotting against N-terminal RIPK1 (leucine 190), middle domain RIPK1 (amino acids 133–422), and GST. **d** Late addition of AG7088 inhibits production of HRV-A16. HeLa cells were infected with HRV-A16 at three different MOI (log −1, −2, −3) and treated with AG7088 at different times pi. The titer of viral progeny was determined from cells and supernatants 24 h pi for each time point by end point titration assays. Data points represent mean values from three different experiments, including SD.

We next characterized the cleavage mechanism of RIPK1. Proteolysis of RIPK1 in puromycin-treated apoptotic cells was suppressed by the pan-caspase inhibitor QVD (as shown in Fig. 5c). Yet, treatments of HRV-A16-infected HeLa cells with QVD, cathepsin/calpain inhibitor E64d or RIPK1-mediated necroptosis inhibitor NEC-1^49^ did not affect the 60 kDa RIPK1 fragment (Fig. 6a). Of note, NEC-1 did not affect RIPK1 cleavage or VP2 production in QVD-treated cells and HRV-A16 infection in hAECN (data not shown), confirming the earlier finding that RIPK1 per se was dispensable for infection.

In stark contrast, the selective 3Cpro inhibitor AG7088 completely abrogated RIPK1 processing, and blocked viral replication as indicated by the lack of VP2 (Fig. 6a). To probe whether the 3Cpro directly cleaved RIPK1, we incubated cell lysates with recombinant 3Cpro (r3C). This led to the appearance of the 60 kDa N-terminal RIPK1 fragment, completely suppressed by AG7088 (Fig. 6b). r3C did not give rise to the 36/37 and 46/47 kDa fragments found in HRV-A16-infected cells, suggesting that other viral or cellular factors generate these fragments. This was confirmed by incubation of recombinant GST-tagged RIPK1 with r3C, which gave rise to a 90 kDa RIPK1 fragment comprising GST, the N-terminal and middle domains of RIPK1, but no 36/37 or 46/47 kDa fragments (Fig. 6c). Notably, appearance of the 90 kDa fragment was inhibited by AG7088 but not by QVD. Remarkably, late addition of AG7088 to HRV-A16-infected cells at 16 h pi clearly reduced the production of infectious virus compared to addition of the drug at 24 h pi (Fig. 6d). At 16 h pi, the amounts of VP0 and VP2 remained constant, implicating that 3Cpro was no longer involved in the processing of VP0 precursor protein (see Fig. 5b). Inhibition of 3Cpro by AG7088 early in infection (prior to onset of virion production) had even stronger effects than late addition, due to inhibition of poly-protein processing. We conclude that 3Cpro cleaves parts of the middle domain and the C-terminus of RIPK1, and enhances virus replication.

### Rhinovirus infection associates 3Cpro with RIPK1 and disrupts death signaling complexes

**Fig. 7:**
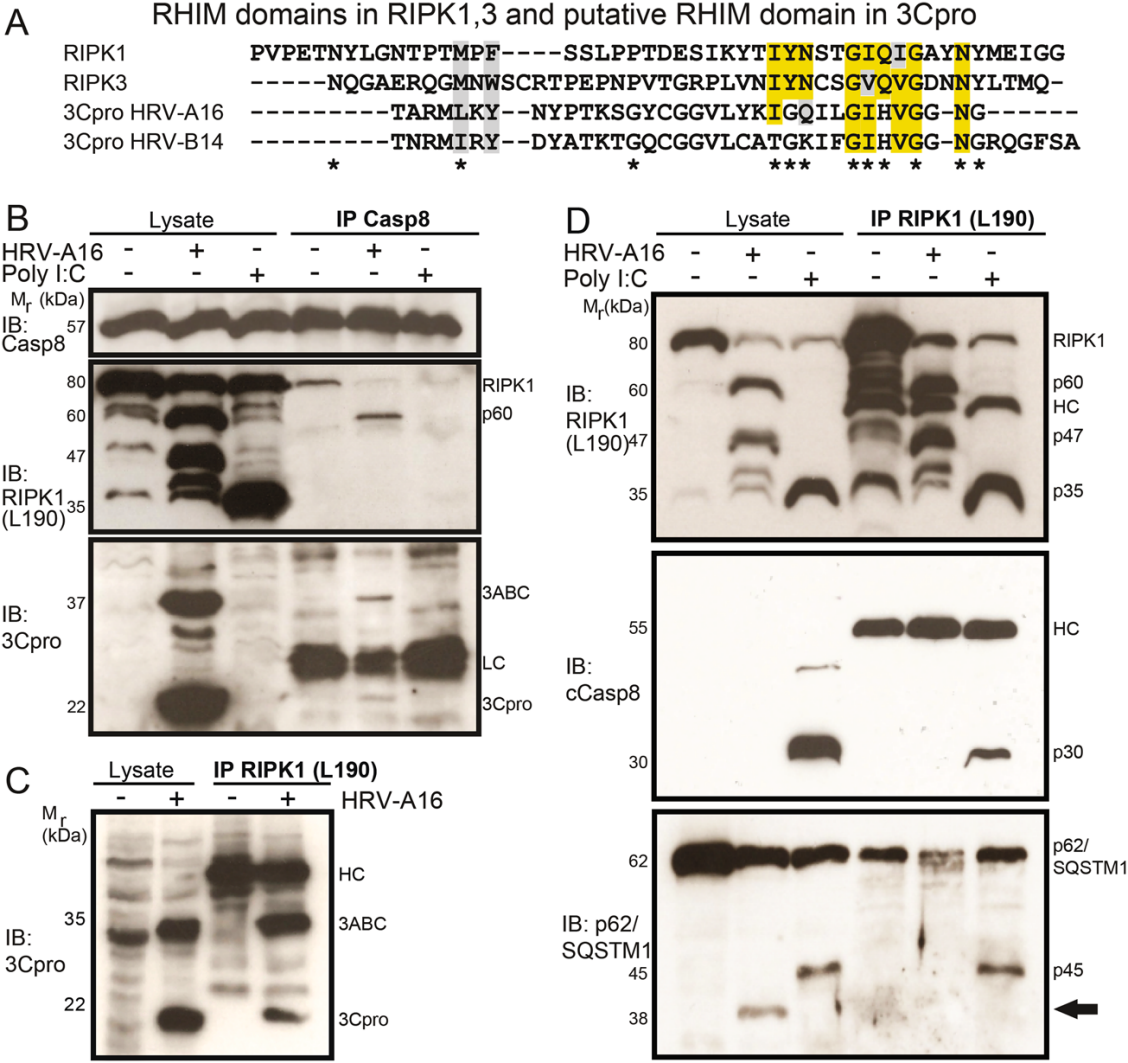
HRV-A16 infection disrupts an RHIM domain-containing RIPK1-cCasp8-p62/SQSTM1 signaling complex, and leads to complex formation of 60 kDa RIPK1-Caspase-8 and 3ABC/3Cpro. **a** Comparison of the RHIM domain from human RIPK1, RIPK3, 3Cpro from HRV-A16/B14 by sequence alignment. Yellow indicates amino acid identities, gray similarities, * denotes conserved amino acids in the RHIM domains of RIPK1/3. **b** Pro-caspase-8 immuno-precipitations, and western blots against Pro-caspase-8, RIPK1 (amino acids 190) and 3Cpro from lysates of HeLa cells infected with HRV-A16 (MOI 1, 15 h), or poly I:C (5 µg/ml)-treated cells, and uninfected samples. Input lysates and immuno-precipitations are shown. LC denotes light chain. **c** RIPK1 immuno-precipitations (leucine 190) and western blot against 3Cpro from HRV-A16-infected HeLa cells (MOI 1, 15 h) or uninfected cells. HC denotes heavy chain. **d** RIPK1 immuno-precipitations (leucine 190) and western blots against RIPK1, cCasp8, and p62/SQSTM1 from lysates of HeLa cells infected with HRV-A16 (MOI 1, 15 h) or transfected with poly I:C (5 µg/ml). Input lysates and immuno-precipitations are shown. HC denotes heavy chain, IP immuno-precipitation. Arrow highlights HRV-A16-specific p62/SQSTM1 processing.

3Cpro of A and B-type HRV (including the viruses under study) may contain a degenerate C-terminal RHIM domain, as defined by evolutionary analyses^50^ (Fig. 7a). RIPK1/3 bind to TRIF via their RHIM domains and transmit signals to caspase-8 activation. We hypothesized that 3Cpro interacts with RIPK1 or TRIF, and thereby cleaves the death kinase RIPK1, and corrupts caspase-8 activation at late stages of infection.

We analyzed RIPK1 immuno-complexes of untreated, poly I:C-treated and HRV-A16-infected cells. Immuno-complexes with anti-procaspase-8 antibodies from untreated cells contained full-length RIPK1 and caspase-8 proteins (Fig. 7b). In contrast, immuno-complexes of poly I:C-activated or HRV-A16-infected cells were devoid of full-length RIPK1. Infected cells contained a complex of pro-caspase-8, p60 N-terminal RIPK1, and the viral 3ABC and 3Cpro proteins. RIPK1 complexes with 3ABC and 3Cpro were confirmed by immuno-precipitation with anti-N-terminal RIPK1 antibodies (Fig. 7c). Further analyses showed that poly I:C-induced RIPK1 fragments were in complex with active p30 caspase-8 and also with a p62/SQSTM1 cleavage product p45 (Fig. 7d). Notably, HRV-A16 infection led to cleavage of p62/SQSTM1 and gave rise to a 38 kDa product. As a consequence, binding of full-length p62/SQSTM1 to RIPK1 was impaired (Fig. 7d). This suggested that binding of 3ABC and 3Cpro to RIPK1-containing immuno-complexes and proteolytic processing of RIPK1 by 3Cpro interfered with death signaling.

**Fig. 8:**
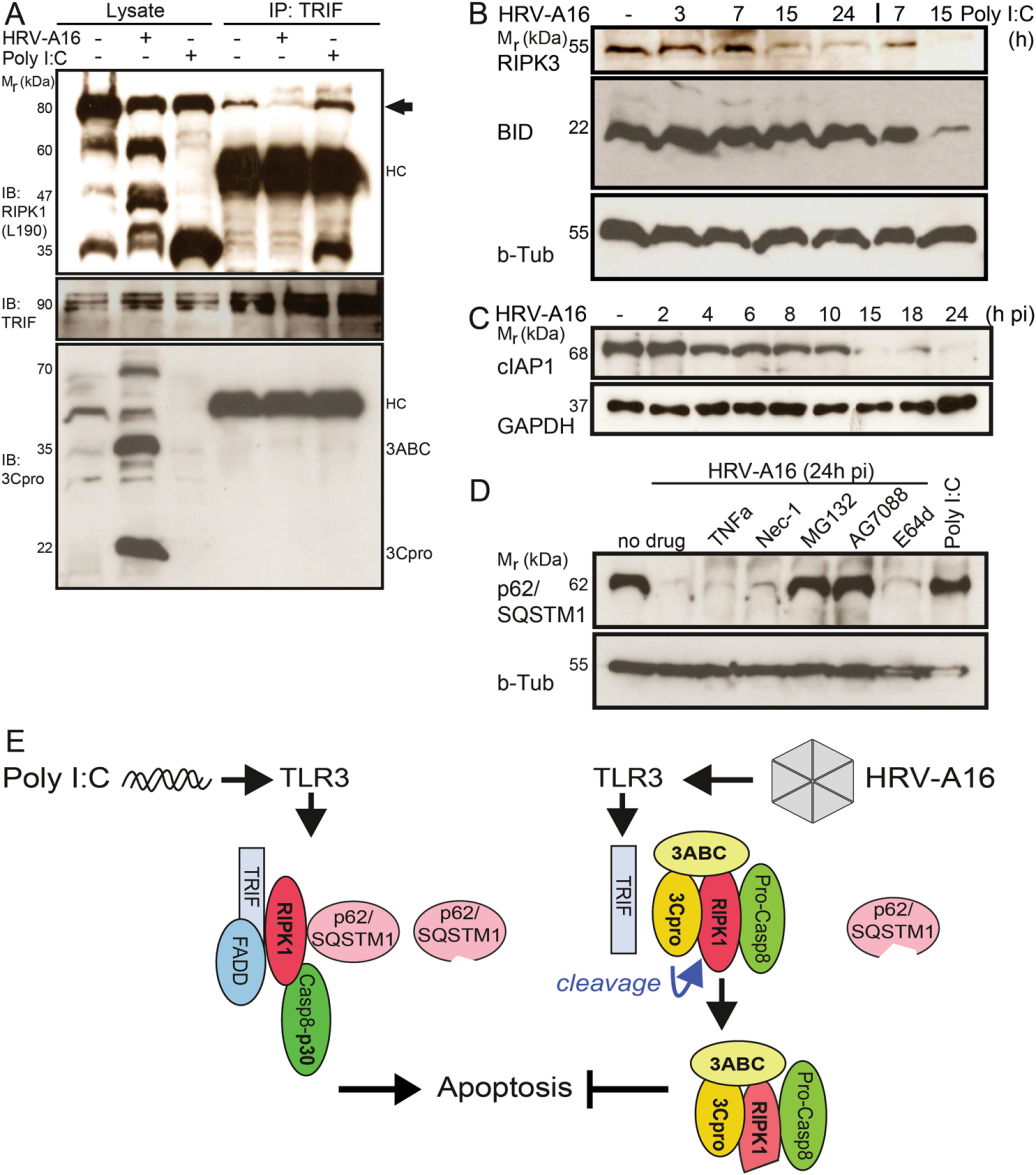
Interruption of RIPK1 death signaling in HRV-A16 infection. **a** Disruption of RIPK1-TRIF complexes shown by anti-TRIF immuno-precipitations, and western blots against TRIF, RIPK1 (amino acids 190), and 3Cpro from lysates of HeLa cells infected with HRV-A16 (MOI 1, 15 h). Poly I:C (5 µg/ml)-transfected control cells did not show TRIF-RIPK1 complex disruption. HC denotes heavy chain, arrow highlights co-precipitated full length RIPK1 from poly I:C-treated cells. **b** Time-resolved degradation of RIPK3 but not BID. Analyses by western blots against RIPK3, BID and beta-tubulin (b-Tub) from total lysates of HeLa cells infected with HRV-A16 (MOI 1) or transfected with poly I:C (5 μg/ml). **c** Time-resolved degradation of cIAP1 in HeLa cells infected with HRV-A16 (MOI 1) analyzed by western blots against cIAP1 and GAPDH. **d** Western blots against p62/SQSTM1 and beta-tubulin from lysates of untreated HeLa cells, or transfected with poly I:C (5 μg/ml), or infected with HRV-A16 (MOI 1, 24 h) ± parallel treatment with TNFα (1 μg/ml), calpain/cathepsin inhibitor E64d (10 μm), necrostatin-1 (Nec1, 1 μm), proteasomal inhibitor MG132 (10 μm) or AG7088 (rupintrivir, 20 nm). p62/SQSTM1 expression is highlighted by black arrow. **e** Schematic depiction of dsRNA-induced death signaling (left) and HRV-A16 interception of death signaling (right). TLR3 senses dsRNA, which leads to formation of TRIF-RIPK1-FADD-p62/SQSTM1 complexes, recruitment, and proteolytic activation of pro-caspase-8 to active p30 caspase-8 (cleaved Casp8) and apoptosis (poly I:C pathway, left). This may involve binding of p30-Casp8 to RIPK1, and cleavage of an N-terminal p35 RIPK1 fragment, and p62/SQSTM1 cleavage. HRV-A16 interferes with this death signaling pathway by 3ABC and 3Cpro binding to RIPK1, and RIPK1 cleavage to an N-terminal 60 kDa fragment. RIPK1 binds to procaspase-8, dissociates from TRIF, FADD, and p62/SQSTM1 and interrupts apoptotic signaling. In addition, degradation of RIPK3, and processing of p62/SQSTM1 (distinct from poly I:C triggered p62/SQSTM1 processing) further attenuate necroptotic and NF-KB signaling. This results in virus controlled necrosis.

TRIF complexes from both control and poly I:C-treated cells contained full-length RIPK1, and in poly I:C-stimulated cells also the proteolytic RIPK1 product p35 (Fig. 8a). This was not observed in HRV-A16-infected cells, although TRIF was abundantly present, akin to FADD, and the complexes lacked full length RIPK1, p60 N-terminal RIPK1, 3ABC, and 3Cpro (not shown). Unlike RIPK1, the TRIF or FADD immuno-complexes did not contain 3ABC or 3Cpro, suggesting that these adaptors are dislocated from the death signaling complex in infected cells.

In addition to C-terminal RIPK1 proteolysis, we found that the levels of RIPK3 and the cellular inhibitor of apoptosis protein 1 (cIAP1) were strongly reduced in HRV-A16-infected epithelial cells 15 h pi (Fig. 8b, c). Depletion of cIAP1 primes cells towards formation of death receptor complex II, also referred to as the ripoptosome, a signaling complex containing RIPK1, RIPK3, FADD, and caspase-8 formed upon genotoxic stress^33^. Further, the depletion of RIPK3 precludes RIPK3 oligomerization and subsequent necrosis and necroptosis. Unlike cIAP1 and RIPK3, BH3 interacting domain death agonist (BID) was not proteolytically processed (Fig. 8b). BID processing is required for activation of the mitochondrial and the extrinsic apoptotic pathways^8^. All these results strengthen the notion that propagation of intrinsic and extrinsic apoptosis by caspases-8/9 is impaired in HRV-A16 infected cells.

We finally investigated the p62/SQSTM1 levels, and found that they were strongly reduced late in HRV-A16 infection but not after poly I:C treatment (Fig. 8d). p62/SQSTM1 is known to interact with RIPK1 and involved in NF-κB activation, caspase-8 recruitment, and protein turnover by autophagy^51^. p62/SQSTM1 depletion was inhibited by the proteasome inhibitor MG132, or the 3Cpro inhibitor AG7088, but not NEC-1, the cathepsin B inhibitor E64d or TNFa (Fig. 8d). NEC-1 blocks RIPK1 kinase activity and death processes, independent of caspases^52^. Collectively, the data show that TRIF-RIPK1 and p62/SQSTM1 containing complexes were assembled in poly I:C-treated cells, and active p30 caspase-8 bound to RIPK1 (Fig. 8e). Apoptotic cleavage fragments of RIPK1 remained bound to TRIF and p62/SQSTM1. Yet, in HRV-A16 infection the RIPK1 complex was substantially altered due to 3Cpro, and consisted of the 60 kDa RIPK1 fragment, procaspase-8, 3ABC and/or active 3Cpro and p62/SQSTM1. We suggest that binding of 3Cpro to RIPK1 gives rise to a death kinase lacking death functions. This corrupts the dsRNA-induced RIPK1-TRIF-FADD complex without affecting TRIF and FADD expression, suggesting that TRIF and FADD may have signaling functions independent of RIPK1.

## Discussion

### A new function for 3Cpro in suppressing apoptosis and necroptosis

How picornaviruses control cell death pathways is poorly known. Here, we explored enterovirus interference with apoptosis and necroptosis execution, and found that 3Cpro controls cell death programs. 3Cpro and its precursors are key enzymes for processing the enteroviral poly-protein. 3Cpro blocks secretion and host transcription, shuts off protein synthesis, and interferes with nucleo-cytoplasmic transport reviewed in refs. ^20,53^. 3Cpro intercepts innate signaling by cleaving a variety of factors, including the TRIF^54,55^, NEMO^56^, the complement component C3^57^, and IPS-1 (interferon beta promoter stimulator protein 1)^17^. 3Cpro also cleaves the stress granule (SG)-associated protein G3BP1, which is essential to recruit protein kinase R (PKR) to SG, and silences protein synthesis^58^.

We show that 3Cpro cleaves the death kinase RIPK1, and precludes apoptotic and necroptotic cell death. This has important implications, since apoptosis and necrosis/necroptosis are well known to antagonize viral infections, and may be involved in restricting virus spread^59^. Our results show that rhinovirus-infected cells suppress the onset of apoptosis and necrosis/necroptosis by expressing the viral 3C protease. This is based on the finding that 3Cpro inhibition by AG7088 reduced virus progeny formation and reduced PI inclusion. Furthermore, the expression of catalytically active 3Cpro per se reduced poly I:C-induced caspase-dependent apoptosis, without itself inducing apoptosis. It enhanced cell lysis, which may boost virus escape from the infected cells and virus spreading to neighboring cells^60–64^.

### 3Cpro removes the death-domain of RIPK1 and impairs apoptosis

This alternative necrosis-like phenotype triggered by 3Cpro appears to involve double-stranded viral RNA and cleavage of RIPK1. dsRNA-triggered apoptosis depends on caspase-8, RIPK1, TRIF, and the RNA-sensor TLR3, but not MDA5 and FADD. Early stages of HRV infection showed no vast signs of apoptosis execution, indicating that incoming viral RNA is not sensed. Late in infection, we found that dsRNA colocalized with TLR3 and RIPK1 in vesicular structures. How dsRNA localizes into these vesicles is unknown, but may involve virus-induced alterations in the infected cell, or dsRNA uptake from nearby lysed cells. Since RIPK1 coprecipitated with a precursor of VP2 and 3Cpro, dsRNA but also viral polyprotein may be sensed. This may initiate apoptosis, as indicated by annexin V and FLICA caspase-8/9 stainings 15 h pi. Yet, the cleavage of RIPK1 by 3Cpro coincided with suppression of apoptosis execution, although we cannot exclude an involvement of 3ABC or 3CD precursors, or other viral proteases affecting alternative RIPK1 processing. Regardless, 3Cpro coimmunoprecipitated with RIPK1, and 3Cpro binding to RIPK1 and cleaving of the RIPK1-DD correlated with the abrogation of signaling from dsRNA-TLR3, and impaired recruitment of active caspase-8, as well as activation of BID and caspase-9. An alternative complex comprising the 60 kDa N-terminal RIPK1 fragment, pro-caspase-8 and 3Cpro was formed, but it lacked activated caspase-8. It is possible that the truncated RIPK1 has intrinsic functions in executing the necrosis-like phenotype, possibly upon further posttranslational modifications and involving host effector proteins. Regardless, the necrosis-like pathway appeared to be independent of the RIPK1 kinase inhibitor NEC-1 or the pan-caspase inhibitor QVD.

### HRV-A16 infection suppresses necroptosis

dsRNA-TLR3 signaling complexes trigger necroptosis, which involves RIPK3/RIPK1 hetero-oligomers, the cleavage of p62/sequestosome and membrane rupture^4,7,37^. Our observations of RIPK3 degradation and cleavage of RIPK1 and p62/SQSTM1 strongly suggest that HRV-A16-infected epithelial cells suppress necroptosis. This raises the possibility that necroptosis can function against RNA viruses, beyond DNA viruses. Neither the RIPK1 kinase inhibitor NEC-1 nor the depletion of RIPK1 by RNAi affected the death pathways or HRV-A16 infection. This suggests that HRV-A16 does not use RIPK1 to execute cell death, but cleaved RIPK1 may affect the stability of RIPK3.

How other dsRNA sensors beyond TLR3 are controlled in enterovirus infections remains to be investigated. Since MDA5 and PKR pathways share adaptor proteins used in TLR3 signaling, it is possible that the MDA5 and PKR pathways are shut-down in HRV infection. Indeed, TLR3 and MDA5, and their adaptors TRIF and MAVS are proteolytically processed by enteroviral and host proteases^19,28,29^. These processes might attenuate NFκB signaling, and production of pro-inflammatory cytokines and type I interferon^31,52^. Together with the impairment of apoptosis and necroptosis found for HRV this indicates that enteroviruses may also be in control of cell death processes. Our results strongly support this notion, and indicate that the proteolytic activity of 3Cpro leads to caspase-independent cell death with features of necrosis.

## Materials and methods

### Cell lines, primary cells, and viruses

HeLa cervical carcinoma cells strain Ohio (HeLa, from L. Kaiser; Central Laboratory of Virology, University Hospital Geneva, Switzerland) were cultured in Dulbecco’s Modified Eagle Medium (DMEM) supplemented with 10% heat-inactivated fetal bovine serum (FBS) and 1% l-glutamine. Human airway epithelial cells from nasal biopsies (hAECN) were cultured as recommended by the supplier (Epithelix, Geneva, Switzerland). HRV-A16 and A1A were provided by W.M. Lee (Department of Pediatrics, School of Medicine and Public Health, University of Wisconsin, USA). HRV-A2, B14, and B37 were provided by L. Kaiser (Central Laboratory of Virology, University Hospital Geneva, Switzerland), and CVB3 by T. Hyypia (Department of Virology, University of Turku, Finland). Infections were at MOI 1, as described^23^, unless indicated otherwise.

### Chemicals and antibodies

Poly I:C was purchased from Sigma, puromycin from Invivogen, z-VAD-fmk from BD Pharmingen, z-LEHD-fmk and z-IETD-fmk from Biovision, QVD-OPH and E-64-d (loxistatin) from SM Biochemicals, TNF-alpha from R&D systems, NEC-1 and MG132 from Tocris and rupintrivir (AG7088) from Axon Medchem. Recombinant RIPK1 protein and HRV3C protease were purchased from SignalChem and Sino Biological Inc., respectively. On TARGETplus SMARTpool siGENOME siRNAs against RIPK1, TLR3, TICAM, FADD, MAVS, MYD88, and MDA5 were purchased from Dharmacon. Antibodies were obtained as follows: mouse monoclonal Mab16-7, and rabbit polyclonal antibody detecting HRV 3C protease were kindly provided by W.M. Lee (Department of Pediatrics, School of Medicine and Public Health, University of Wisconsin, USA) used as in ref. ^23^ and S. Amineva (Department of Pediatrics, School of Medicine and Public Health, University of Wisconsin, USA), respectively. Mab J2 (English & Scientific Consulting). Mabs against RIPK1 (C-12, aa 465–671), RIPK3 (Rippy-3), caspase-8, beta-tubulin, and rabbit polyclonal antibody against lamin A/C (Santa Cruz Biotechnology), Mab anti-p62/SQSTM1 (MBL), rabbit polyclonal antibodies against RIPK1 (D94C12, aa190), FADD, TRIF, MDA5, BID, cleaved PARP, caspases-9, -7, -3, cleaved caspases-8, -7, -3, GAPDH and HRP-linked secondary antibodies against mouse or rabbit IgG (Cell Signaling), rabbit polyclonal antibody against GST (Sigma), rabbit polyclonal antibody against cIAP1, TLR3 and RIPK1 (aa165–402) (Abcam), Mab against RIPK1 (aa133–422) (Origene), and Alexa Fluor-488 or -594 labeled secondary antibodies against mouse or rabbit IgG (Invitrogen). Immunogold-conjugated secondary antibodies (goat anti-mouse IgG 20 nm, goat anti-rabbit IgG 10 nm) were purchased from BBI Solutions.

### High-throughput infection of hAECN

hAECN were cultured in 96-well plates as recommended by the supplier (Epithelix, Geneva, Switzerland). At a confluence of 10,000 cells/well, cells were infected with purified HRVs or CV at different MOIs in hAECN culture medium at 33.5 °C for 24 h, fixed in 4% paraformaldehyde (PFA) and permeabilized with 0.2% Triton X-100. Immunostaining was performed with mabJ2 antibody diluted in PBS containing 1% bovine serum albumin (BSA) as described previously^23^. Images were acquired with an ImageXpress Micro microscope (Molecular Devices) in automated mode, using a CoolSNAP HQ 12 bit gray scale camera (Roper Scientific), ×10/NA 0.5 objective (Nikon) and analyzed with a custom written script in Matlab (MathWorks, Inc. Natick, MA, USA). Infection indexes (fraction of infected cells per total cell number) were plotted with GraphPad Prism software (GraphPad).

### Virus titration

HeLa cells were seeded in 24-well plates, cultured overnight at 37 °C in full medium, infected with purified HRV-A16 (MOI1) in infection medium in presence or absence of pan-caspase inhibitor z-VAD-fmk (20 μm). At various time points post-infection, supernatant and adherent cultures were collected. Cell-associated virus from adherent cultures was released by three freeze- and thaw steps and subjected together with cell supernatant to titrations by serial tenfold dilutions in 96-well plates containing nearly confluent HeLa cells. Cells were incubated for 4 days at 33.5 °C, fixed and stained with crystal violet. The median tissue culture infectious dose (TCID_50_) was calculated according to the Spearman−Kaerber formula.

### RNA interference and virus mRNA transfection

siRNAs (20 nm, Dharmacon pools) were reverse transfected to HeLa cells in 24-well or opaque 96-well plates using serum-free Opti-MEM (Invitrogen) and Lipofectamine RNAiMAX (Invitrogen) according to the manufacturer’s protocol. Seventy-two hours post-transfection, cells were infected with purified HRV-A16 at MOI 1 in infection medium (DMEM supplemented with 0.2% BSA, 1% l-glutamine and 30 mm MgCl_2_) or alternatively with poly I:C (5 μg/ml) at 33.5 °C for 15 h in presence or absence of inhibitors. Cells in 96-well plates were subjected to cell viability (Resazurin) assays and cells in 24-well plates were analyzed by western blot analysis. For entry by-pass, HeLa cells were grown in 12-well plates in full medium at 37 °C for 24 h and transfected with viral RNA extracted from purified HRV-A16 (TransIT-mRNA transfection kit, Mirus Bio, USA). At different time points post transfection cells were lysed in total lysis buffer (62.5 mm Tris, pH 6.8; 2% SDS [v/v]; 10% glycerol [v/v]; 6 m urea; 5% b-mercaptoethanol [v/v]; 0.01% bromophenolblue [w/v]; 0.01% phenolred [w/v]) and subjected to western blot analysis.

### Transfection of GFP-3Cpro expression constructs

Plasmids expressing N-terminal GFP fusion constructs of catalytically active or inactive 3Cpro were kindly provided by Reena Ghildyal (Monash University, Clayton, Australia), described elsewhere^65^. Constructs were transfected into HeLa cells in 12-well dishes at a density of 350,000 cells per well using Lipofectamine 3000 (Invitrogen) according to the manufacturer’s protocol. DMEM medium supplemented with 10% heat-inactivated FBS and 1% l-glutamine was replaced by serum-free Opti-MEM (Invitrogen) 24 h post-transfection, and poly I:C (5 μg/ml) was transfected into cells using Lipfectamine RNAiMAX (Invitrogen). Adherent cells were analyzed by annexin V-APC/propidium iodide staining 24 h post transfection.

### Cell viability assay

In order to determine cell viability/metabolic activity, 20 μl of Resazurin solution (0.15 mg/ml in PBS, pH 7.4) was added to siRNA-, drug-treated or infected HeLa cells in opaque-walled 96-well plates to a final volume of 120 μl/well and incubated for 1 h at 37 °C. Fluorescence was recorded with a M200 Infinite Plate Reader (Tecan) using a 540 nm excitation/590 nm emission filter set. Relative fluorescence units (RFU) were plotted with GraphPad Prism software (GraphPad).

### Reactive oxygen species detection

To detect reactive oxygen species produced late during the course of infection HeLa cells or hAECN in 96-well plates were infected with HRV-A16 or HRV-A1A at an MOI 1 for 15 or 24 h, respectively, and incubated for 1 h at 37 °C with CellROX Green Reagent (Molecular Probes) at a final concentration of 5 μm. Cells were washed three times with PBS and fixed with 3.7% formaldehyde in PBS. Menadione (100 μm) and Luprox (100 μm) served as positive controls for ROS production. Fluorescence was recorded with an M200 Infinite Plate Reader (Tecan) using a 485 nm excitation/520 nm emission filter set. RFU were plotted with GraphPad Prism software (GraphPad).

### Annexin/propidium iodide staining and caspase activity assays (FLICA)

For both assays cells were cultivated in six-well plates at a density of 5×10^5^ cells/ well and infected the next day at an MOI 1. At various timepoints post-infection apoptosis or necrosis of adherent cells was determined using annexin V-FITC (Miltenyi Biotec) and annexin V-APC (Biolegend) kits according to the manufacturer’s instructions. Caspase-8 and 9 activities were analyzed using carboxyfluorescein FLICA caspase assays (Immunochemistry Technologies, LCC) according to the instructions of the manufacturer. Puromycin (5 μg/ml) and poly I:C (5 μg/ml) served as positive controls for apoptosis. Fluorescence was measured within an hour after staining by flow cytometry. For each sample, 10,000 events were analyzed using an Epics Elite ESP (Beckman Coulter), MACSQuant10 analyzer, and WinMDI 2.8 or Flowlogic software. The fractions of positive cells were plotted with GraphPad Prism software (GraphPad).

### DNA fragmentation assay

HeLa cells in 10 cm dishes were scratched and pelleted for 5 min at 220 g. After PBS washing the pellet was dissolved in DNA laddering buffer (5 mm Tris, pH 7.4; 20 mm EDTA; 0.5% Triton X-100) on ice to lyse the cells. To pellet the chromatin lysates were centrifuged at 4 °C and 10,000 g for 15 min. The supernatant containing DNA fragments was phenol/chloroform purified and re-extracted by chloroform addition. The DNA fragments containing phase was ethanol-precipitated and ethanol-washed pellets were dissolved in DNase- and RNase-free water (Gibco) for 30 min at RT. To remove RNA contents RNase A (10 mg/ml, DNase free) was added to the samples for 30 min at RT. Samples were separated by gel electrophoresis (1.2% agarose gels containing EtBr in TAE buffer) at 45 V for 4 h and analyzed by UV transilluminator (G:BOX UV transilluminator, Syngene).

### In vitro protease cleavage assays

Confluent monolayers of HeLa cells were lysed in cold RIPA buffer (50 mm Tris, pH 8.0, 150 mm sodium chloride, 1.0% NP-40, 0.5% sodium deoxycholate, 0.1% sodium dodecyl sulfate) for 1 h on ice, followed by centrifugation at 10,000 g for 1 min to remove cell debris. Lysates equivalent to 1.0×10^6^ cells were incubated with four units of recombinant HRV 3C protease (Sino Biological Inc) at 4 °C for 16 h in presence or absence of inhibitors. Samples were heated at 100 °C for 5 min in Laemmli buffer to stop the reaction, followed by western blot analysis. Alternatively, 2 μg of recombinant RIPK1 protein in 50 mm Tris, pH 8.0, 150 mm sodium chloride was incubated as described above with recombinant 3C protease with or without inhibitors.

### Western blots

For protein expression analysis after siRNA treatments, poly I:C treatment or transfection, drug treatment and/ or infection, HeLa cell or hAECN cells in 24-well plates were washed with PBS and directly lysed in total lysis buffer (62.5 mm Tris, pH 6.8; 2% SDS [v/v]; 10% glycerol [v/v]; 6 m urea; 5% b-mercaptoethanol [v/v]; 0.01% bromophenol-blue [w/v]; 0.01% phenolred [w/v]) on ice. Samples were separated by SDS-PAGE, transferred to Hybond-P PVDF membranes (GE Healthcare) and blocked in TBS-T buffer containing 5% milk or BSA for 1 h. Primary antibodies were incubated overnight at 4 °C following incubation with horseradish peroxidase-conjugated secondary antibodies for 1 h at RT. Signals were detected using ECL Plus (GE Healthcare). For immuno-precipitation analysis of RIPK1 containing complexes untreated, HRV-A16-infected or poly I:C-transfected HeLa cells in 10 cm dishes were collected by scratching and washed with PBS. 1/10 of each sample was lysed in total lysis buffer. 9/10 of the washed and pelleted sample was lysed in 150 mm NaCl and 1% Triton-X-100 in 20 mm Tris, pH 8.0, containing protease inhibitor cocktail (Roche). Lysates were pre-absorbed by incubation with protein A- or G-sepharose (Amersham) at 4 °C for 1 h. To carry out coimmunoprecipitation specific antibody was added to the cleared lysate overnight at 4 °C followed by protein A- or G-sepharose incubation for 90 min at 4 °C. Samples were washed four times with lysis buffer and once with 10 mm Tris, pH 8.0 and subjected to western blot analysis as described above.

### Electron microscopy

HeLa cells or hAECN on coverslips were infected with HRV-A16 at an MOI 1, poly I:C (5 μg/ml) transfected or puromycin (5 μg/ml) treated for 15 h. For transmission electron microscopy cells were fixed with 2% paraformaldehyde/1.5% glutaraldehyde in 0.1 m sodium cacodylate buffer, followed by 1% osmium tetroxide and 1% potassium ferricyanide treatment. Aceton-dried and epon embedded cells were cut in 90 nm slices and images were taken using a TEM10 (Zeiss) with an Erlangshen ES 55W model 782 camera at 80 kV. Cryo-sections of 80 nm thickness were prepared from fixed cells with an Ultracut EM UC6/FC6 (Leica), and immunogold labeled as described previously^66,67^.

### Immunofluorescence and confocal microscopy

HeLa cells or hAECN on coverslips were infected with HRV-A16 at an MOI 1 for 24 h. For immunostainings, cells were fixed with 4% PFA and permeabilized with 0.2% Triton X-100, blocked for 1 h in PBS supplemented with 1% BSA, followed by incubation with primary antibodies in blocking buffer overnight at 4 °C. Alexa Fluor-488 or -594 secondary antibodies were diluted in blocking buffer for 1 h. Coverslips were mounted in mounting medium (Dako) and analyzed with an inverted Leica TCS SP2 scanning laser confocal microscope with an HCX PL APO ×63/1.4 oil immersion objective. Alternatively, images were acquired by Leica SP8, ×63/1.40 oil (HC PL APO CS2), zoom ×3, speed 400 Hz, line average and resolution 4, using LAS AF software (Leica) and processed with ImageJ (National Institutes of Health).

### Statistics

The figures were assembled using Adobe Photoshop and Illustrator. Graphs represent mean values of analyzed samples (*n*) including the SD and *p*values from one-way ANOVA with post-hoc Tukey HSD test.

## Electronic supplementary material

Supplementary Figures S1-S4

## References

[1] Galluzzi L (2012). Molecular definitions of cell death subroutines: recommendations of the Nomenclature Committee on Cell Death 2012. Cell Death Differ..

[2] Mocarski ES, Upton JW, Kaiser WJ (2012). Viral infection and the evolution of caspase 8-regulated apoptotic and necrotic death pathways. Nat. Rev. Immunol..

[3] Ferguson TA, Choi J, Green DR (2011). Armed response: how dying cells influence T-cell functions. Immunol. Rev..

[4] Newton K, Manning G (2016). Necroptosis and inflammation. Annu. Rev. Biochem..

[5] Hengartner MO (2000). The biochemistry of apoptosis. Nature.

[6] Strasser A, O’Connor L, Dixit VM (2000). Apoptosis signaling. Annu. Rev. Biochem..

[7] Estornes Y (2012). dsRNA induces apoptosis through an atypical death complex associating TLR3 to caspase-8. Cell Death Differ..

[8] Galluzzi L, Brenner C, Morselli E, Touat Z, Kroemer G (2008). Viral control of mitochondrial apoptosis. PLoS Pathog..

[9] Connolly PF, Fearnhead HO (2017). Viral hijacking of host caspases: an emerging category of pathogen-host interactions. Cell Death Differ..

[10] Li C (2017). Foot-and-mouth disease virus induces lysosomal degradation of host protein kinase PKR by 3C proteinase to facilitate virus replication. Virology.

[11] Agol VI (2000). Competing death programs in poliovirus-infected cells: commitment switch in the middle of the infectious cycle. J. Virol..

[12] Agol VI (1998). Two types of death of poliovirus-infected cells: caspase involvement in the apoptosis but not cytopathic effect—competing death programs in poliovirus-infected cells: commitment switch in the middle of the infectious cycle. Virology.

[13] Buenz EJ, Howe CL (2006). Picornaviruses and cell death. Trends Microbiol..

[14] Carthy CM (2003). Bcl-2 and Bcl-xL overexpression inhibits cytochrome c release, activation of multiple caspases, and virus release following coxsackievirus B3 infection. Virology.

[15] Belov GA (2003). The major apoptotic pathway activated and suppressed by poliovirus. J. Virol..

[16] Deszcz L, Gaudernak E, Kuechler E, Seipelt J (2005). Apoptotic events induced by human rhinovirus infection. J. Gen. Virol..

[17] Drahos J, Racaniello VR (2009). Cleavage of IPS-1 in cells infected with human rhinovirus. J. Virol..

[18] Mukherjee A (2011). The coxsackievirus B 3C protease cleaves MAVS and TRIF to attenuate host type I interferon and apoptotic signaling. PLoS Pathog..

[19] Qu L (2011). Disruption of TLR3 signaling due to cleavage of TRIF by the hepatitis A virus protease-polymerase processing intermediate, 3CD. PLoS Pathog..

[20] Laitinen OH (2016). Enteroviral proteases: structure, host interactions and pathogenicity. Rev. Med. Virol..

[21] Busse WW, Lemanske RF, Gern JE (2010). Role of viral respiratory infections in asthma and asthma exacerbations. Lancet.

[22] Kennedy JL, Turner RB, Braciale T, Heymann PW, Borish L (2012). Pathogenesis of rhinovirus infection. Curr. Opin. Virol..

[23] Jurgeit A (2010). An RNA replication-center assay for high content image-based quantifications of human rhinovirus and coxsackievirus infections. Virol. J..

[24] Roulin PS (2014). Rhinovirus uses a phosphatidylinositol 4-phosphate/cholesterol counter-current for the formation of replication compartments at the ER−Golgi interface. Cell Host Microbe.

[25] Belov GA (2016). Dynamic lipid landscape of picornavirus replication organelles. Curr. Opin. Virol..

[26] Hewson CA, Jardine A, Edwards MR, Laza-Stanca V, Johnston SL (2005). Toll-like receptor 3 is induced by and mediates antiviral activity against rhinovirus infection of human bronchial epithelial cells. J. Virol..

[27] Wang Q (2009). Role of double-stranded RNA pattern recognition receptors in rhinovirus-induced airway epithelial cell responses. J. Immunol..

[28] Feng Q (2012). MDA5 detects the double-stranded RNA replicative form in picornavirus-infected cells. Cell Rep..

[29] Harris KG, Coyne CB (2013). Enter at your own risk: how enteroviruses navigate the dangerous world of pattern recognition receptor signaling. Cytokine.

[30] Rui Y (2017). Disruption of MDA5-mediated innate immune responses by the 3C proteins of Coxsackievirus A16, Coxsackievirus A6, and Enterovirus D68. J. Virol..

[31] Festjens N, Vanden Berghe T, Cornelis S, Vandenabeele P (2007). RIP1, a kinase on the crossroads of a cell’s decision to live or die. Cell Death Differ..

[32] Biton S, Ashkenazi ANEMO (2011). and RIP1 control cell fate in response to extensive DNA damage via TNF-alpha feedforward signaling. Cell.

[33] Tenev T (2011). The Ripoptosome, a signaling platform that assembles in response to genotoxic stress and loss of IAPs. Mol. Cell.

[34] Zhang DW (2009). RIP3, an energy metabolism regulator that switches TNF-induced cell death from apoptosis to necrosis. Science.

[35] He S (2009). Receptor interacting protein kinase-3 determines cellular necrotic response to TNF-alpha. Cell.

[36] Silke J, Rickard JA, Gerlic M (2015). The diverse role of RIP kinases in necroptosis and inflammation. Nat. Immunol..

[37] Goodall ML (2016). The autophagy machinery controls cell death switching between apoptosis and necroptosis. Dev. Cell.

[38] Feoktistova M (2011). cIAPs block Ripoptosome formation, a RIP1/caspase-8 containing intracellular cell death complex differentially regulated by cFLIP isoforms. Mol. Cell.

[39] Jurgeit A (2012). Niclosamide is a proton carrier and targets acidic endosomes with broad antiviral effects. PLoS Pathog..

[40] Croons V, Martinet W, Herman AG, De Meyer GRY (2008). Differential effect of the protein synthesis inhibitors puromycin and cycloheximide on vascular smooth muscle cell viability. J. Pharmacol. Exp. Ther..

[41] Sanwal V (2001). Puromycin aminonucleoside induces glomerular epithelial cell apoptosis. Exp. Mol. Pathol..

[42] Binford SL (2005). Conservation of amino acids in human rhinovirus 3C protease correlates with broad-spectrum antiviral activity of rupintrivir, a novel human rhinovirus 3C protease inhibitor. Antimicrob. Agents Chemother..

[43] Hoffmann JC, Pappa A, Krammer PH, Lavrik IN (2009). A new C-terminal cleavage product of procaspase-8, p30, defines an alternative pathway of procaspase-8 activation. Mol. Cell. Biol..

[44] Rodrigue-Gervais IG, Saleh M (2013). Caspases and immunity in a deadly grip. Trends Immunol..

[45] Kaczmarek A, Vandenabeele P, Krysko DV (2013). Necroptosis: the release of damage-associated molecular patterns and its physiological relevance. Immunity.

[46] Thakar J, Schleinkofer K, Borner C, Dandekar T (2006). RIP death domain structural interactions implicated in TNF-mediated proliferation and survival. Proteins.

[47] Orr DC, Long AC, Kay J, Dunn BM, Cameron JM (1989). Hydrolysis of a series of synthetic peptide substrates by the human rhinovirus 14 3C proteinase, cloned and expressed in Escherichia coli. J. Gen. Virol..

[48] Blom N, Hansen J, Blaas D, Brunak S (1996). Cleavage site analysis in picornaviral polyproteins: discovering cellular targets by neural networks. Protein Sci..

[49] Degterev A (2008). Identification of RIP1 kinase as a specific cellular target of necrostatins. Nat. Chem. Biol..

[50] Kajava AV, Klopffleisch K, Chen S, Hofmann K (2014). Evolutionary link between metazoan RHIM motif and prion-forming domain of fungal heterokaryon incompatibility factor HET-s/HET-s. Sci. Rep..

[51] Jin Z (2009). Cullin3-based polyubiquitination and p62-dependent aggregation of caspase-8 mediate extrinsic apoptosis signaling. Cell.

[52] Ofengeim D, Yuan J (2013). Regulation of RIP1 kinase signalling at the crossroads of inflammation and cell death. Nat. Rev. Mol. Cell Biol..

[53] Sun D, Chen S, Cheng A, Wang M (2016). Roles of the picornaviral 3C proteinase in the viral life cycle and host cells. Viruses.

[54] Lei X (2011). Cleavage of the adaptor protein TRIF by enterovirus 71 3C inhibits antiviral responses mediated by Toll-like receptor 3. J. Virol..

[55] Xiang Z (2014). Enterovirus 68 3C protease cleaves TRIF to attenuate antiviral responses mediated by Toll-like receptor 3. J. Virol..

[56] Wang D (2012). Foot-and-mouth disease virus 3C protease cleaves NEMO to impair innate immune signaling. J. Virol..

[57] Tam JC, Bidgood SR, McEwan WA, James LC (2014). Intracellular sensing of complement C3 activates cell autonomous immunity. Science.

[58] Reineke LC, Lloyd RE (2015). The stress granule protein G3BP1 recruits protein kinase R to promote multiple innate immune antiviral responses. J. Virol..

[59] Thomson BJ (2001). Viruses and apoptosis. Int. J. Exp. Pathol..

[60] Prasad V (2014). Chemical induction of unfolded protein response enhances cancer cell killing through lytic virus infection. J. Virol..

[61] Yakimovich A (2015). Plaque2.0—a high-throughput analysis framework to score virus-cell transmission and clonal cell expansion. PLoS ONE.

[62] Yakimovich, A. et al. Infectio: a generic framework for computational simulation of virus transmission between cells. *mSphere* **1**, e00078-15 (2016). 10.1128/mSphere.00078-15 PMC4863613 27303704

[63] Yakimovich, A. et al. Inhibition of poxvirus gene expression and genome replication by bisbenzimide derivatives. *J. Virol*. **91**, e00838-17(2017). 10.1128/JVI.00838-17 PMC5571260 28659488

[64] Greber UF, Bartenschlager R (2017). Editorial: an expanded view of viruses. Fems Microbiol. Rev..

[65] Walker E (2016). Rhinovirus 16 2A protease affects nuclear localization of 3CD during infection. J. Virol..

[66] Tokuyasu KT (1973). A technique for ultracryotomy of cell suspensions and tissues. J. Cell. Biol..

[67] Luisoni, S. et al. Endosomophagy clears disrupted early endosomes but not virus particles during virus entry into cells. *Matters**,* DOI: 10.19185/matters.201606000013, 1−33 (2016).

