# Supplementary material for: Rhinovirus 3C protease suppresses apoptosis and triggers caspase-independent cell death
Source: Cell Death Dis. 2018 Feb 15;9(3):272. doi: 10.1038/s41419-018-0306-6 (PMC5833640; doi:10.1038/s41419-018-0306-6)

Supplementary Information

Supplementary Figure S1 (related to Figure 1): Poly I:C or puromycin induced apoptosis (annexin V-positive) is inhibited by QVD or zVADfmk but not NEC-1, and poly I:C or puromycin induced propidium iodide-positive cells are enhanced by QVD but not by NEC-1.

A) FACS analysis of annexin V staining of HeLa cells transfected with poly I:C and treated with QVD (5 and 10 μM), z-VADfmk (10, 50 and 100 μM), NEC-1 (10 and 50 μM) or AG7088 (10 and 20 μM). Values are means (n=3); error bars represent the SD. * indicates significant differences to poly I:C with $P<0.01$.

B) FACS analysis of annexin V and propidium iodide (PI) stainings of HeLa cells treated with puromycin in presence of inhibitors (QVD, 10 μM; NEC-1, 50 μM). Values are means (n=3); error bars represent the SD. * indicates significant differences to puromycin with $P<0.01$.

C) FACS analysis of PI staining of HeLa cells transfected with poly I:C and treated with QVD (5 and 10 μM), z-VADfmk (10, 50 and 100 μM), NEC-1 (10 and 50 μM) and AG7088 (10 and 20 μM). Values are means (n=3); error bars represent the SD. * indicates significant differences to poly I:C with $P<0.01$.

D) FACS analysis of annexin V staining of HeLa cells treated with puromycin in presence of inhibitors (QVD, 10 μM; NEC-1, 50 μM). Values are means (n=3); error bars represent the SD. * indicates significant differences to puromycin with $P<0.01$.

E) Western blots against cleaved caspase-8, VP2, 3Cpro and GAPDH from lysates of untreated HeLa cells, or cells transfected with poly I:C (5 μg/ml) or infected with HRV-A16 for 15h, +/- NEC-1 (50 μM), QVD (10 μM) or both inhibitors.

SF1

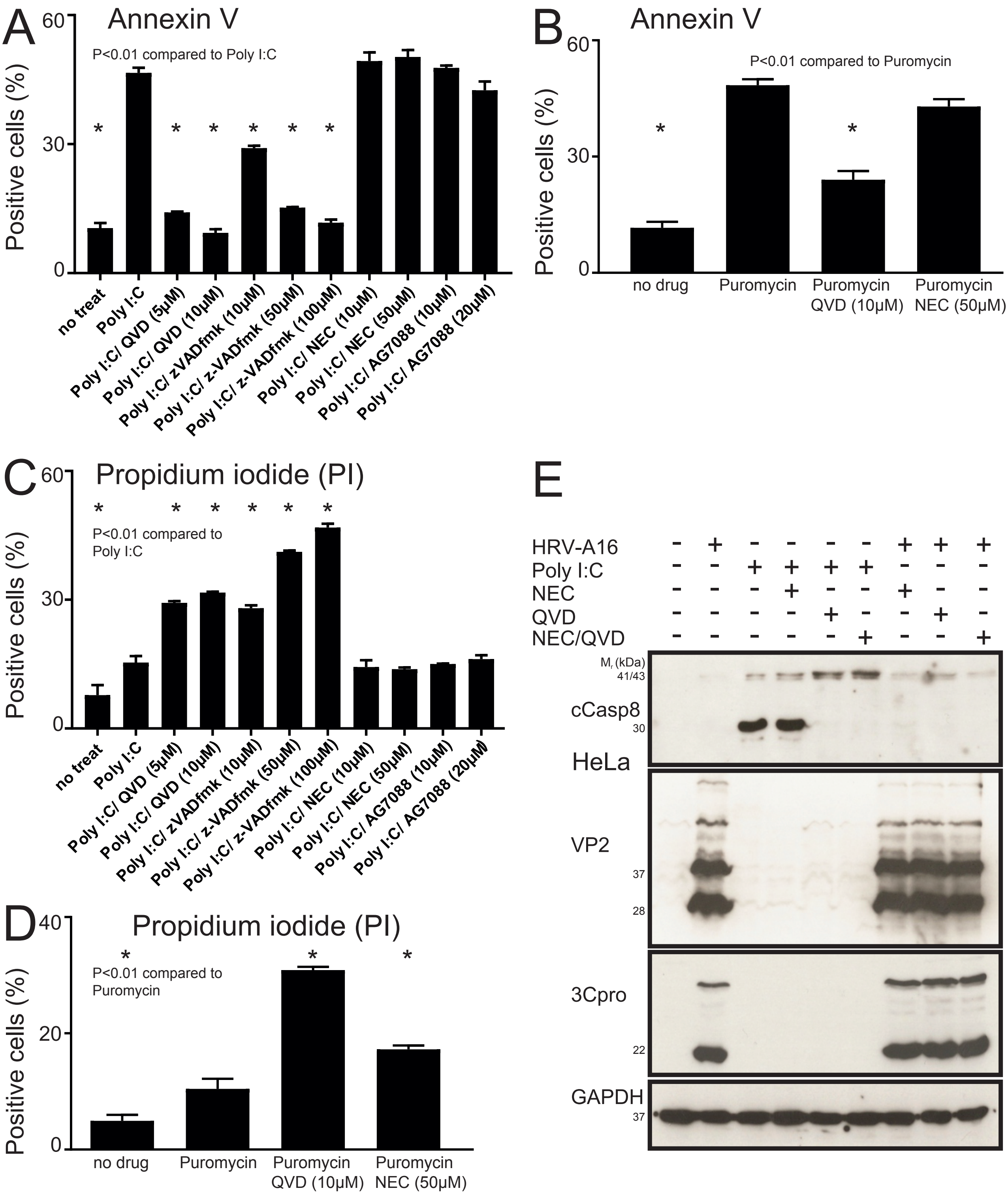
A
Annexin V
P<0.01 compared to Poly I:C
Positive cells (%)
60
30
0
no treat
Poly I:C
Poly I:C/ QVD (5µM)
Poly I:C/ QVD (10µM)
Poly I:C/ zVADfmk (10µM)
Poly I:C/ z-VADfmk (50µM)
Poly I:C/ z-VADfmk (100µM)
Poly I:C/ NEC (10µM)
Poly I:C/ NEC (50µM)
Poly I:C/ AG7088 (10µM)
Poly I:C/ AG7088 (20µM)
B
Annexin V
P<0.01 compared to Puromycin
Positive cells (%)
no drug
Puromycin
Puromycin QVD (10µM)
Puromycin NEC (50µM)
C
Propidium iodide (PI)
P<0.01 compared to Poly I:C
Positive cells (%)
D
Propidium iodide (PI)
P<0.01 compared to Puromycin
Positive cells (%)
40
20
0
no drug
Puromycin
Puromycin QVD (10µM)
Puromycin NEC (50µM)
E
HRV-A16
Poly I:C
NEC
QVD
NEC/QVD
Mr (kDa)
41/43
cCasp8
30
HeLa
VP2
37
28
3Cpro
22
GAPDH
37

Supplementary Figure S2 (related to Figure 3): Puromycin-induced caspase-8/9 activities are blocked by the broad inhibitor QVD or specific inhibitors (panel A), and poly I:C-induced caspase-8/9 activities are blocked by HRV-A16 or QVD (panels B, C).

A) FLICA caspase-8/9 activity assays in HeLa cells treated with puromycin for 15h in presence of caspase inhibitors (QVD, 10 µM; z-IETDfmk, 100 µM and z-LEHDfmk, 100 µM). Values are means (n=3); error bars represent the SD. * indicates significant differences to puromycin with P<0.01.

B) FLICA caspase-8/9 activity assays in HeLa cells treated with poly I:C or infected with HRV-A16 (MOI1, 15h) and puromycin treated cells (9h). Values are means (n=3); error bars represent the SD. * indicates significant differences to puromycin with P<0.01.

C) FLICA caspase-8/9 activity assays in HeLa cells treated with poly I:C or infected with HRV-A16 (MOI1) and transfected with poly I:C (6h pi) +/- the broad caspase inhibitor QVD (10 µM). Values are means (n=3); error bars represent the SD. * indicates significant differences to poly I:C with P<0.01.

SF2

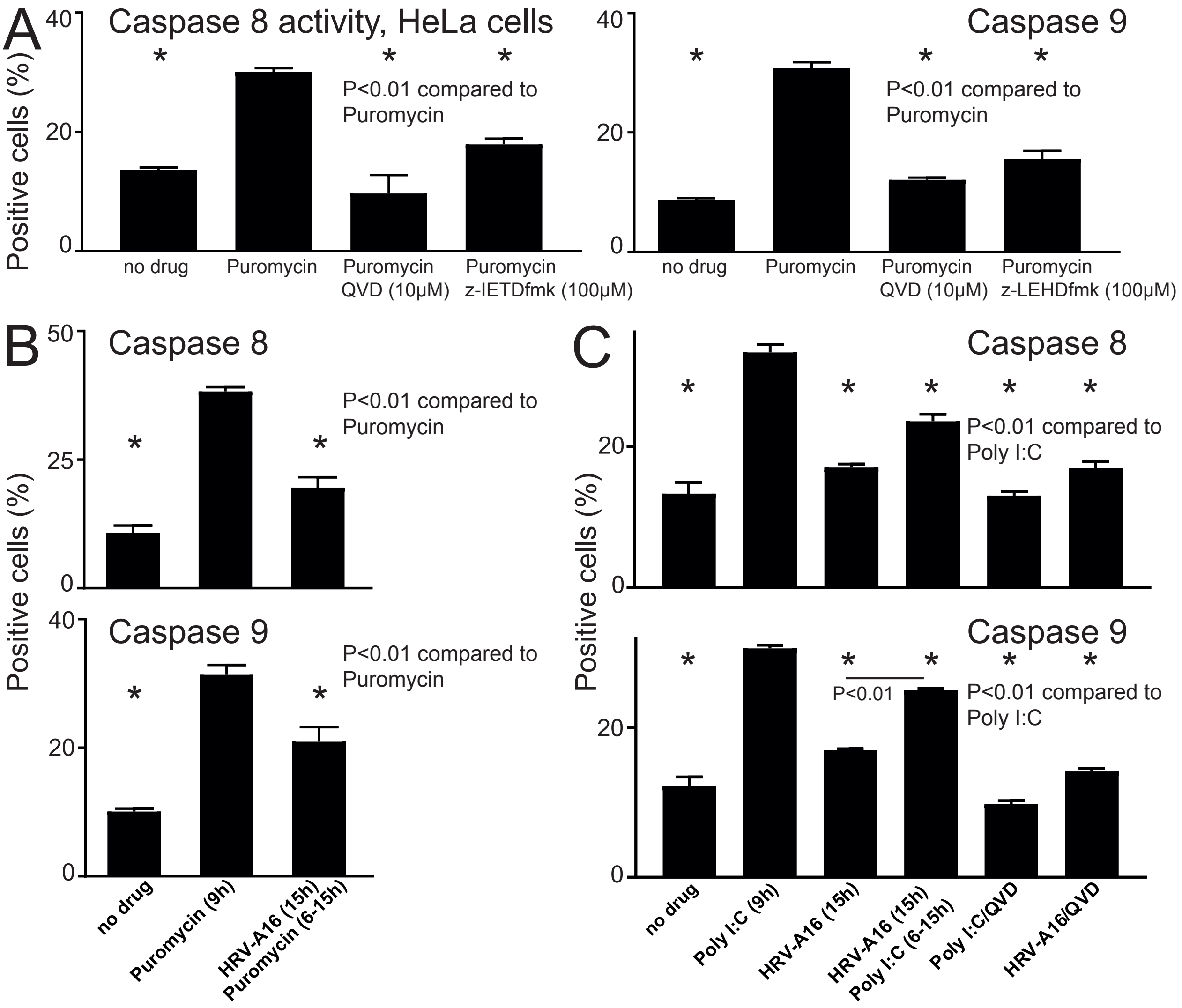
A
Caspase 8 activity, HeLa cells
Caspase 9
Positive cells (%)
P<0.01 compared to Puromycin
no drug
Puromycin
Puromycin QVD (10μM)
Puromycin z-IETDfmk (100μM)
Puromycin z-LEHDfmk (100μM)
B
Caspase 8
Caspase 9
no drug
Puromycin (9h)
HRV-A16 (15h) Puromycin (6-15h)
C
P<0.01 compared to Poly I:C
P<0.01
Poly I:C (9h)
HRV-A16 (15h)
HRV-A16 (15h) Poly I:C (6-15h)
Poly I:C/QVD
HRV-A16/QVD

Supplementary Figure S3 (related to Figure 3): HRV-A16 infection suppresses poly I:C-induced apoptosis (annexin V positive), and enhances viral necrosis (propidium iodide positive).

A) FACS analysis of annexin V staining of HeLa cells infected with HRV-A16 (MOI1, 15h) and transfected with poly I:C (0h or 6h pi). Values are means (n=3); error bars represent the SD; $P<0.01$ (one-way ANOVA with post-hoc Tukey HSD test).

B) Western blots against cleaved caspase-8 (cCasp8), cleaved caspase-3 (cCasp3), VP2, 3Cpro, cleaved PARP (cPARP) and b-tubulin from lysates of untreated or HRV-A16 infected HeLa cells (15h pi), or transfected with poly I:C (5 µg/ml) for 15h.

C) FACS analysis of propidium iodide (PI) staining of HeLa cells infected with HRV-A16 (MOI1, 15h) and transfected with poly I:C (0h pi or 6h pi). Values are means (n=3); error bars represent the SD; $P<0.01$ (one-way ANOVA with post-hoc Tukey HSD test).

D) FACS analysis of PI staining of HeLa cells infected with HRV-A16 (MOI1, 15h) and treated with QVD (10 µM), NEC-1 (50 µM), or AG7088 (20 µM), and comparison to poly I:C transfected cells. Values are means (n=3); error bars represent the SD.

SF3

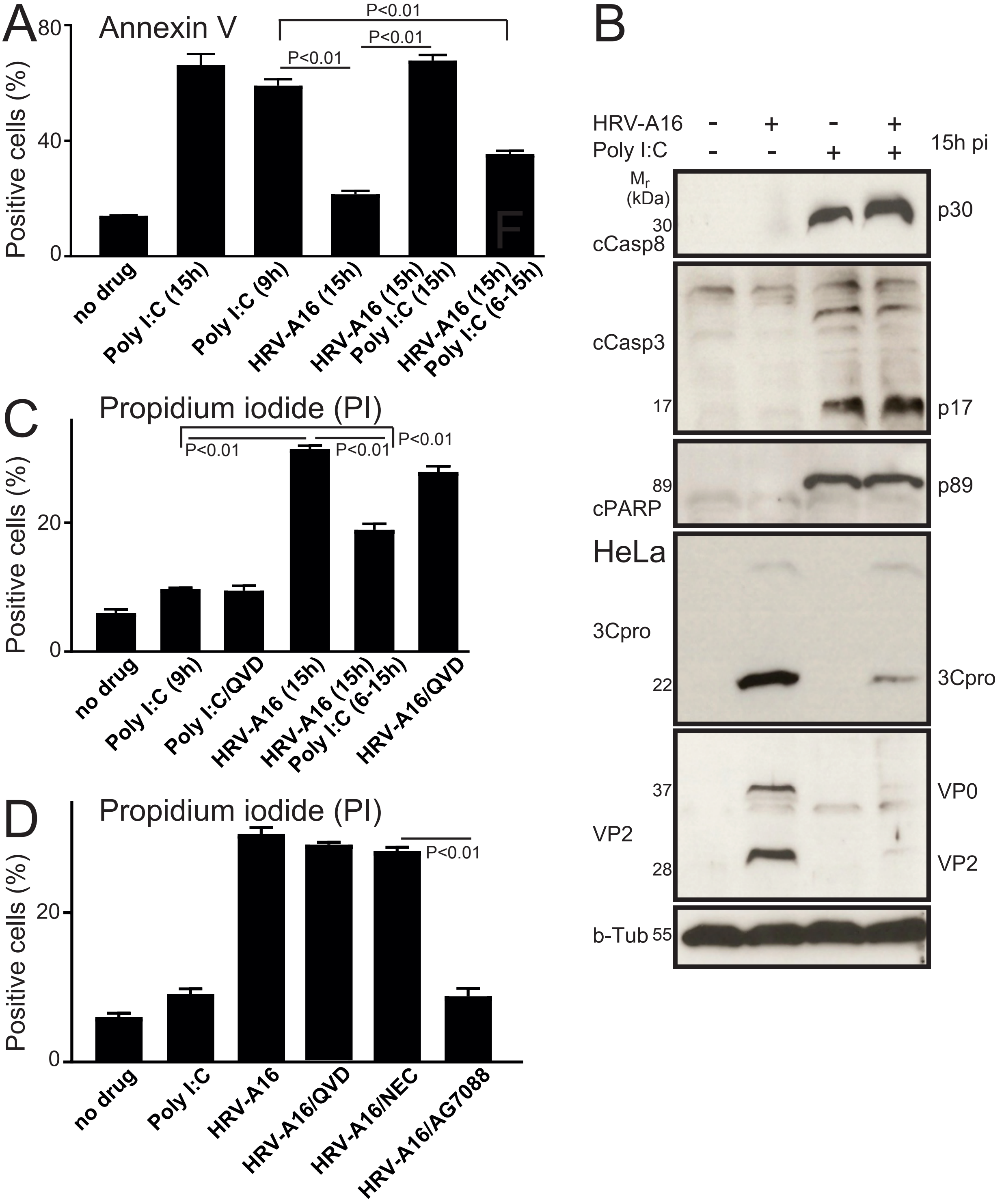
A
Annexin V
Positive cells (%)
80
40
0
P<0.01
P<0.01
P<0.01
no drug
Poly I:C (15h)
Poly I:C (9h)
HRV-A16 (15h)
HRV-A16 (15h)
Poly I:C (15h)
HRV-A16 (15h)
Poly I:C (6-15h)
B
HRV-A16
Poly I:C
15h pi
M_r
(kDa)
30
cCasp8
p30
cCasp3
17
p17
89
cPARP
p89
HeLa
3Cpro
22
3Cpro
37
VP0
VP2
28
VP2
b-Tub
55
C
Propidium iodide (PI)
Positive cells (%)
20
0
P<0.01
P<0.01
P<0.01
no drug
Poly I:C (9h)
Poly I:C/QVD
HRV-A16 (15h)
HRV-A16 (15h)
Poly I:C (6-15h)
HRV-A16/QVD
D
Propidium iodide (PI)
Positive cells (%)
20
0
P<0.01
no drug
Poly I:C
HRV-A16
HRV-A16/QVD
HRV-A16/NEC
HRV-A16/AG7088

Supplementary Figure S4 (related to Figure 3G): HRV-A16 3Cpro suppresses poly I:C mediated apoptosis (annexin V positive), and induces necrosis (propidium iodide positive).

HeLa cells were transfected with 1 μg of catalytically active or inactive GFP-tagged 3C protease (3C-pro) for 24 h, followed by transfection with poly I:C (5 μg/ml, 15h) and annexin V and prodium iodide assays. Values are means (n=3); error bars represent the SD; $P<0.01$ (one-way ANOVA with post-hoc Tukey HSD test).

SF4

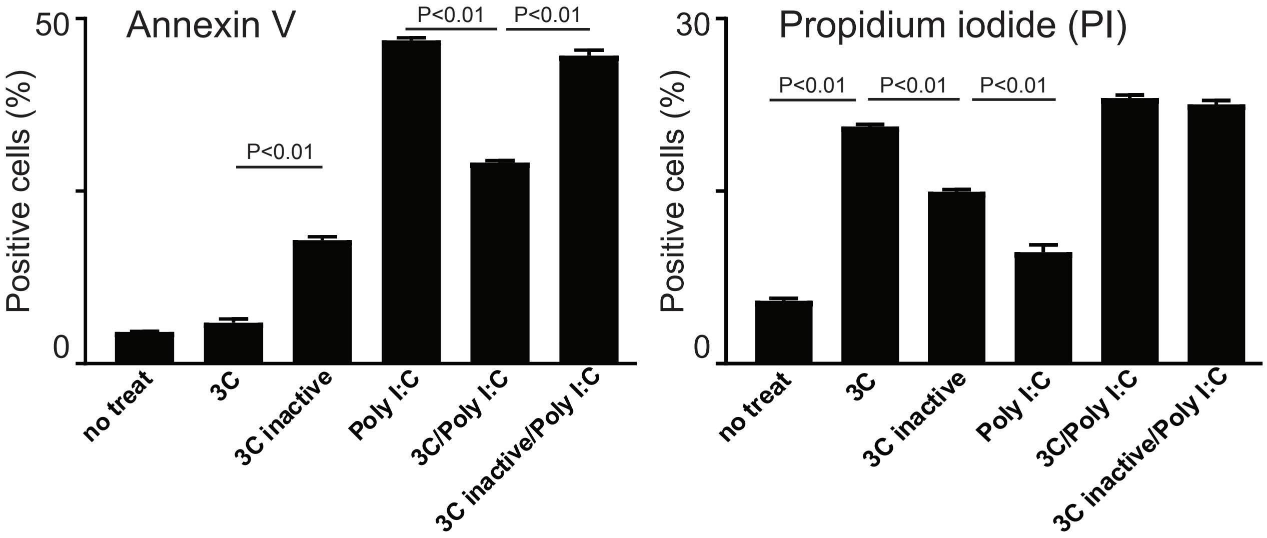

Supplement: Supplementary file 1 — Supplementary Figures S1-S4 [file 41419_2018_306_MOESM1_ESM.pdf]
